# Systematic evaluation and external validation of 22 prognostic models among hospitalised adults with COVID-19: an observational cohort study

**DOI:** 10.1183/13993003.03498-2020

**Published:** 2020-12-24

**Authors:** Rishi K. Gupta, Michael Marks, Thomas H.A. Samuels, Akish Luintel, Tommy Rampling, Humayra Chowdhury, Matteo Quartagno, Arjun Nair, Marc Lipman, Ibrahim Abubakar, Maarten van Smeden, Wai Keong Wong, Bryan Williams, Mahdad Noursadeghi

**Affiliations:** 1 Institute for Global Health, University College London, London, UK; 2 University College London Hospitals NHS Trust, London, UK; 3 Clinical Research Dept, Faculty of Infectious and Tropical Diseases, London School of Hygiene and Tropical Medicine, London, UK; 4 MRC Clinical Trials Unit, Institute of Clinical Trials and Methodology, University College London, London, UK; 5 UCL Respiratory, Division of Medicine, University College London, London, UK; 6 Julius Center for Health Sciences and Primary Care, University Medical Center Utrecht, Utrecht University, Utrecht, The Netherlands; 7 NIHR University College London Hospitals Biomedical Research Centre, London, UK; 8 University College London, London, UK; 9 Division of Infection and Immunity, University College London, London, UK; 10 Members of the UCLH COVID-19 Reporting Group are listed in the acknowledgements section

## Abstract

The number of proposed prognostic models for coronavirus disease 2019 (COVID-19) is growing rapidly, but it is unknown whether any are suitable for widespread clinical implementation.

We independently externally validated the performance of candidate prognostic models, identified through a living systematic review, among consecutive adults admitted to hospital with a final diagnosis of COVID-19. We reconstructed candidate models as per original descriptions and evaluated performance for their original intended outcomes using predictors measured at the time of admission. We assessed discrimination, calibration and net benefit, compared to the default strategies of treating all and no patients, and against the most discriminating predictors in univariable analyses.

We tested 22 candidate prognostic models among 411 participants with COVID-19, of whom 180 (43.8%) and 115 (28.0%) met the endpoints of clinical deterioration and mortality, respectively. Highest areas under receiver operating characteristic (AUROC) curves were achieved by the NEWS2 score for prediction of deterioration over 24 h (0.78, 95% CI 0.73–0.83), and a novel model for prediction of deterioration <14 days from admission (0.78, 95% CI 0.74–0.82). The most discriminating univariable predictors were admission oxygen saturation on room air for in-hospital deterioration (AUROC 0.76, 95% CI 0.71–0.81), and age for in-hospital mortality (AUROC 0.76, 95% CI 0.71–0.81). No prognostic model demonstrated consistently higher net benefit than these univariable predictors, across a range of threshold probabilities.

Admission oxygen saturation on room air and patient age are strong predictors of deterioration and mortality among hospitalised adults with COVID-19, respectively. None of the prognostic models evaluated here offered incremental value for patient stratification to these univariable predictors.

## Abstract 2

**Oxygen saturation on room air and patient age are strong predictors of deterioration and mortality, respectively, among hospitalised adults with COVID-19. None of the 22 prognostic models evaluated in this study adds incremental value to these univariable predictors.** https://bit.ly/2Hg24TO

## Introduction

Coronavirus disease 2019 (COVID-19), caused by severe acute respiratory syndrome coronavirus-2 (SARS-CoV-2), causes a spectrum of disease ranging from asymptomatic infection to critical illness. Among people admitted to hospital, COVID-19 has a reported mortality of 21%–33%, with 14%–17% requiring admission to high dependency or intensive care units (ICUs) [1–4]. Exponential surges in transmission of SARS-CoV-2 coupled with the severity of disease among a subset of those affected pose major challenges to health services by threatening to overwhelm resource capacity [5]. Rapid and effective triage at the point of presentation to hospital is therefore required to facilitate adequate allocation of resources and to ensure that patients at higher risk of deterioration are managed and monitored appropriately. Prognostic models may have additional value in patient stratification for emerging drug therapies [6, 7].

As a result, there has been global interest in developing prediction models for COVID-19 [8]. These include models aiming to predict a diagnosis of COVID-19, and prognostic models aiming to predict disease outcomes. At the time of writing, a living systematic review has already catalogued 145 diagnostic or prognostic models for COVID-19 [8]. Critical appraisal of these models using quality assessment tools developed specifically for prediction modelling studies suggests that the candidate models are poorly reported and at high risk of bias and overestimation of their reported performance [8, 9]. However, independent evaluation of candidate prognostic models in unselected datasets has been lacking. It remains unclear how well these proposed models perform in practice, or whether any are suitable for widespread clinical implementation. We aimed to address this knowledge gap by systematically evaluating the performance of proposed prognostic models among consecutive patients hospitalised with a final diagnosis of COVID-19 at a single centre when using predictors measured at the point of hospital admission.

## Methods

### Identification of candidate prognostic models

We used a published living systematic review to identify all candidate prognostic models for COVID-19 indexed in PubMed, Embase, Arxiv, medRxiv or bioRxiv until May 5, 2020, regardless of underlying study quality [8]. We included models that aim to predict clinical deterioration or mortality among patients with COVID-19. We also included prognostic scores commonly used in clinical practice [10–12], but not specifically developed for COVID-19 patients, given that these models may also be considered for use by clinicians to aid risk-stratification for patients with COVID-19. For each candidate model identified, we extracted predictor variables, outcome definitions (including time horizons), modelling approaches and final model parameters from original publications. We contacted authors for additional information where required. We excluded scores for which the underlying model parameters were not publicly available, because we were unable to reconstruct them, along with models for which included predictors were not available in our dataset. The latter included models that require computed tomography imaging or arterial blood gas sampling, because these investigations were not routinely performed among unselected patients with COVID-19 at our centre.

### Study population

Our study is reported in accordance with transparent reporting of a multivariable prediction model for individual prognosis or diagnosis (TRIPOD) guidance for external validation studies [13]. We included consecutive adults admitted to University College Hospital London with a final diagnosis of PCR-confirmed (including all sample types) or clinically diagnosed COVID-19, between February 1 and April 30, 2020. Because we sought to use data from the point of hospital admission to predict outcomes, we excluded patients transferred in from other hospitals and those with hospital-acquired COVID-19 (defined as the first PCR swab sent >5 days from date of hospital admission, as a proxy for the onset of clinical suspicion of SARS-CoV-2 infection). Clinical COVID-19 diagnoses were made on the basis of manual record review by an infectious disease specialist, using clinical features, laboratory results and radiological appearances, in the absence of an alternative diagnosis. During the study period, PCR testing was performed on the basis of clinical suspicion, and no SARS-CoV-2 serology investigations were routinely performed.

### Data sources and variables of interest

Data were collected by direct extraction from electronic health records, complemented by manual curation. Variables of interest in the dataset included demographics (age, sex, ethnicity), comorbidities (identified through manual record review), clinical observations, laboratory measurements, radiology reports and clinical outcomes. Each chest radiograph was reported by a single radiologist, who was provided with a short summary of the indication for the investigation at the time of request, reflecting routine clinical conditions. Chest radiographs were classified using British Society of Thoracic Imaging criteria, and using a modified version of the Radiographic Assessment of Lung Edema (RALE) score [14, 15]. For each predictor, measurements were recorded as part of routine clinical care. Where serial measurements were available, we included the measurement taken closest to the time of presentation to hospital, with a maximum interval between presentation and measurement of 24 h.

### Outcomes

For models that used ICU admission or death, or progression to “severe” COVID-19 or death, as composite endpoints, we used a composite “clinical deterioration” endpoint as the primary outcome. We defined clinical deterioration as the initiation of ventilatory support (continuous positive airway pressure, non-invasive ventilation, high-flow nasal cannula oxygen, invasive mechanical ventilation or extracorporeal membrane oxygenation) or death, equivalent to World Health Organization (WHO) Clinical Progression Scale ≥6 [16]. This definition does not include standard oxygen therapy. We did not apply any temporal limits on 1) the minimum duration of respiratory support or 2) the interval between presentation to hospital and the outcome. The rationale for this composite outcome was to make the endpoint more generalisable between centres, given that hospital respiratory management algorithms may vary substantially. Defining the outcome based on level of support, as opposed to ward setting, also ensured that it is appropriate in the context of a pandemic, when treatments that would usually only be considered in an ICU setting may be administered in other environments owing to resource constraints. Where models specified their intended time horizon in their original description, we used this timepoint in the primary analysis to ensure unbiased assessment of model calibration. Where the intended time horizon was not specified, we assessed the model to predict in-hospital deterioration or mortality, as appropriate. All deterioration and mortality events were included, regardless of their clinical aetiology.

Participants were followed up clinically to the point of discharge from hospital. We extended follow-up beyond discharge by cross-checking National Health Service (NHS) Spine records to identify reported deaths post-discharge, thus ensuring >30 days’ follow-up for all participants.

### Statistical analyses

For each prognostic model included in the analyses, we reconstructed the model according to the authors’ original descriptions, and sought to evaluate the model discrimination and calibration performance against our approximation of their original intended endpoint. For models that provide online risk calculator tools, we validated our reconstructed models against original authors’ models by cross-checking our predictions against those generated by the web-based tools for a random subset of participants.

For all models, we assessed discrimination by quantifying the area under the receiver operating characteristic curve (AUROC) [17]. For models that provided outcome probability scores, we assessed calibration by visualising calibration of predicted *versus* observed risk using LOESS-smoothed plots, and by quantifying calibration slopes and calibration-in-the-large (CITL). A perfect calibration slope should be 1; slopes <1 indicate that risk estimates are too extreme, while slopes >1 reflect risk estimates not being extreme enough. Ideal CITL is 0; CITL>0 indicates that predictions are systematically too low, while CITL<0 indicates that predictions are too high. For models with points-based scores, we assessed calibration visually by plotting model scores *versus* actual outcome proportions. For models that provide probability estimates, but where the model intercept was not available, we calibrated the model to our dataset by calculating the intercept when using the model linear predictor as an offset term, leading to perfect CITL. This approach, by definition, overestimated calibration with respect to CITL, but allowed us to examine the calibration slope in our dataset.

We also assessed the discrimination of each candidate model for standardised outcomes of 1) our composite endpoint of clinical deterioration and 2) mortality across a range of pre-specified time horizons from admission (7 days, 14 days, 30 days and any time during hospital admission) by calculating time-dependent AUROCs (with cumulative sensitivity and dynamic specificity) [18]. The rationale for this analysis was to harmonise endpoints to facilitate more direct comparisons of discrimination between the candidate models.

To further benchmark the performance of candidate prognostic models, we then computed AUROCs for a limited number of univariable predictors considered to be of highest importance *a priori*, based on clinical knowledge and existing data, for predicting our composite endpoints of clinical deterioration and mortality (7 days, 14 days, 30 days and any time during hospital admission). The *a priori* predictors of interest examined in this analysis were age, clinical frailty scale, oxygen saturation at presentation on room air, C-reactive protein level and absolute lymphocyte count [8, 19].

Decision curve analysis allows the clinical utility of candidate models to be assessed, and is dependent on both model discrimination and calibration [20]. We performed decision curve analyses to quantify the net benefit achieved by each model for predicting the original intended endpoint across a range of risk:benefit ratios [20]. In this approach, the risk:benefit ratio is analogous to the cut point for a statistical model above which an intervention or treatment would be considered beneficial (deemed the “threshold probability”). Net benefit was calculated as sensitivity×prevalence–(1–specificity)×(1–prevalence)×w where w is the odds at the threshold probability and the prevalence is the proportion of patients who experienced the outcome [20]. We calculated net benefit across a range of clinically relevant threshold probabilities, ranging from 0 to 0.5, because the risk:benefit ratio may vary for any given intervention. We compared the utility of each candidate model against strategies of treating all and no patients, and against the best-performing univariable predictor for in-hospital clinical deterioration, or mortality, as appropriate. To ensure that fair, head-to-head net benefit comparisons were made between multivariable probability-based models, points score models and univariable predictors, we calibrated each of these to the validation dataset for the purpose of decision curve analysis. Probability-based models were recalibrated to the validation data by refitting logistic regression models with the candidate model linear predictor as the sole predictor. We calculated “delta” net benefit as net benefit when using the index model minus net benefit when 1) treating all patients and 2) using the most discriminating univariable predictor. Decision curve analyses were done using the *rmda* package in R [21].

We handled missing data using multiple imputation by chained equations [22], using the *mice* package in R [23]. All variables and outcomes in the final prognostic models were included in the imputation model to ensure compatibility [22]. A total of 10 imputed datasets were generated; discrimination, calibration and net benefit metrics were pooled using Rubin's rules [24].

All analyses were conducted in R (version 3.5.1; R Foundation for Statistical Computing, Vienna, Austria).

### Sensitivity analyses

We recalculated discrimination and calibration parameters for each candidate model using 1) a complete case analysis (in view of the large amount of missingness for some models), 2) excluding patients without PCR-confirmed SARS-CoV-2 infection and 3) excluding patients who met the clinical deterioration outcome within 4 h of arrival at hospital. We also examined for non-linearity in the *a priori* univariable predictors using restricted cubic splines, with three knots. Finally, we estimated optimism for discrimination and calibration parameters for the *a priori* univariable predictors using bootstrapping (1000 iterations), using the *rms* package in R [25].

### Ethical approval

The pre-specified study protocol was approved by East Midlands - Nottingham 2 Research Ethics Committee (REF: 20/EM/0114; IRAS: 282900).

## Results

### Summary of candidate prognostic models

We identified 37 studies describing prognostic models, of which 19 studies (including 22 unique models) were eligible for inclusion (supplementary figure S1 and table 1). Of these, five models were not specific to COVID-19, but were developed as prognostic scores for emergency department attendees [27], hospitalised patients [12, 44], people with suspected infection [10] or community-acquired pneumonia [11]. Of the 17 models developed specifically for COVID-19, most (10 out of 17) were developed using datasets originating in China. Overall, discovery populations included hospitalised patients and were similar to the current validation population with the exception of one study that discovered a model using community data [28], and another that used simulated data [29]. A total of 13 out of 22 models use points-based scoring systems to derive final model scores, with the remainder using logistic regression modelling approaches to derive probability estimates. A total of 12 out of 22 prognostic models had the primary aim of predicting clinical deterioration, while the remaining 10 sought to predict mortality alone. When specified, time horizons for prognosis ranged from 1 to 30 days. Candidate prognostic models not included in the current validation study are summarised in supplementary table S1.

**TABLE 1:** Characteristics of studies describing prognostic models included in systematic evaluation

**Authors**	**Score name**	**Country of derivation**	**Development population**	**Pre-existing or COVID-specific?**	**Model outcome**	**Predictors**	**Original modelling approach**	**How are predictors combined?**
**Subbe*et al.* [26]**	MEWS^#^	UK	Hospital inpatients	Pre-existing (hospital patients)	Mortality, ICU admission or cardiac arrest (no specified timepoint)	Systolic blood pressure, pulse rate, respiratory rate, temperature, AVPU score	Clinical consensus	Points-based score
**Olsson*et al.* [27]**	REMS^#^	Sweden	Patients presenting to emergency department	Pre-existing (emergency department patients)	Mortality (in-hospital)	Blood pressure, respiratory rate, pulse rate, Glasgow coma scale, oxygen saturation, age	Logistic regression	Points-based score
**Seymour*et al.* [10]**	qSOFA	USA	Electronic health record encounters	Pre-existing (suspected infection)	Mortality (in-hospital)	Systolic hypotension (≤100 mmHg, tachypnoea (≥22 beats·min^−1^), altered mentation	Logistic regression	Points-based score
**Lim*et al.* [11]**	CURB65	UK, New Zealand, Netherlands	Patients with community-acquired pneumonia	Pre-existing (community-acquired pneumonia)	Mortality (30 days)	Confusion, urea >7 mmol·L^−1^, respiratory rate >30 breaths·min^−1^, low systolic (<90 mmHg) or diastolic (<60 mmHg) blood pressure, age >65 years	Logistic regression	Points-based score
**Royal College of Physicians [12]**	NEWS2^+^	UK	Hospital admissions	Pre-existing (hospital patients)	Mortality, ICU admission or cardiac arrest (24 h)	Respiratory rate, oxygen saturation, systolic blood pressure, pulse rate, level of consciousness or new confusion, temperature	Clinical consensus	Points-based score
**Bello-Chavolla*et al.* [28]**	BelloChavolla	Mexico	Confirmed COVID-19 patients presenting in primary care	COVID-specific	Mortality (30 day)	Age ≥65 years, diabetes, early-onset diabetes, obesity, age <40 years, chronic kidney disease, hypertension, immunosuppression (rheumatoid arthritis, lupus, HIV or immunosuppressive drugs)	Cox regression	Points-based score
**Caramelo*et al.* [29]**	Caramelo^¶^	Simulated data	Simulated data	COVID-specific	Mortality (period unspecified)	Age, hypertension, diabetes, CVD, chronic respiratory disease, cancer	Logistic regression	Logistic regression
**Carr*et al.* [30]**	Carr_final, Carr_threshold	UK	Inpatients with confirmed COVID-19	COVID-specific	ICU admission or death (14 days from symptom onset)	NEWS2, CRP, neutrophils, estimated glomerular filtration rate, albumin, age	Regularised logistic regression with LASSO estimator	Regularised logistic regression
**Colombi*et al.* [31]**	Colombi_clinical^¶^ (clinical model only)	Italy	Inpatients with confirmed COVID-19	COVID-specific	ICU admission or in-hospital mortality (period unspecified)	Age >68 years, CVD, CRP >76 mg·L^−1^, LDH >347 U·L^−1^, platelets >180×10^9^ L^−1^	Logistic regression	Logistic regression
**Galloway*et al.* [32]**	Galloway	UK	Inpatients with confirmed COVID-19	COVID-specific	ICU admission or death during admission	Modified RALE score >3, oxygen saturation <93%, creatinine >100 μmol·L^−1^, neutrophils >8×10^9^ L^−1^, age >40 years, chronic lung disease, CRP >40 mg·L^−1^, albumin <34 g·L^−1^, male, non-white ethnicity, hypertension, diabetes	Logistic regression (LASSO)	Points-based score
**Guo*et al.* [33]**	Guo	China	Inpatients with confirmed COVID-19	COVID-specific	Deterioration within 14 days of admission	Age >50 years, underlying chronic disease (not defined), neutrophil/lymphocyte ratio >5, CRP >25 mg·L^−1^, D-dimer >800 ng·mL^−1^	Cox regression	Points-based score
**Hall*et al.* [34]**	TACTIC	UK	Inpatients with confirmed COVID-19	COVID-specific	Admission to ICU or death during admission	Modified RALE score >3, age >40 years, male, non-white ethnicity, diabetes, hypertension, neutrophils >8×10^9^ L^−1^, CRP >40 mg·L^−1^	Logistic regression (LASSO)	Points-based score
**Hu*et al.* [35]**	Hu	China	Inpatients with confirmed COVID-19	COVID-specific	Mortality (in-hospital)	Age, CRP, lymphocytes, D-dimer (μg/mL)	Logistic regression	Logistic regression
**Huang*et al.* [36]**	Huang	China	Inpatients with confirmed COVID-19	COVID-specific	Progression to severe COVID (defined as respiratory rate ≥30 breaths·min^−1^, oxygen saturation ≤93% in the resting state or *P*_aO_2__/oxygen concentration FiO_2_ ≤300 mmHg), 3–7 days from admission	CRP >10 mg·L^−1^, LDH >250 U·L^−1^, respiratory rate >24 breaths·min^−1^, comorbidity (hypertension, coronary artery disease, diabetes, obesity, COPD, chronic kidney disease, obstructive sleep apnoea)	Logistic regression	Logistic regression
**Ji*et al.* [37]**	Ji	China	Inpatients with confirmed COVID-19	COVID-specific	Progression to severe COVID-19 at 10 days (defined as respiratory rate ≥30 breaths·min^−1^, resting oxygen saturation ≤93%, *P*_aO_2__/FiO_2_ ≤300 mmHg, requirement of mechanical ventilation or worsening of lung CT findings)	Age >60 years, lymphocytes ≤1×10^9^ L^−1^) LDH <250, 250–500, >500 U·L^−1^, comorbidity (hypertension, diabetes, CVD, chronic lung disease or HIV)	Cox regression	Points-based score
**Lu*et al.* [38]**	Lu	China	Inpatients with suspected or confirmed COVID-19	COVID-specific	Mortality (12 days)	Age ≥60 years, CRP ≥34 mg·L^−1^	Cox regression	Points-based score
**Shi*et al.* [39]**	Shi	China	Inpatients with confirmed COVID-19	COVID-specific	Death or “severe” COVID-19 (not defined) over unspecified period	Age >50 years, male, hypertension	Not specified	Points-based score
**Xie*et al.* [40]**	Xie	China	Inpatients with confirmed COVID-19	COVID-specific	Mortality (in-hospital)	Age, lymphocytes, LDH, oxygen saturation	Logistic regression	Logistic regression
**Yan*et al.* [41]**	Yan	China	Inpatients with suspected COVID-19	COVID-specific	Mortality (period unspecified)	LDH >365 U·L^−1^, CRP >41.2 mg·L^−1^, lymphocyte percentage >14.7%	Decision-tree model with XG boost	Points-based score
**Zhang*et al.* [42]**	Zhang_poor, Zhang_death	China	Inpatients with confirmed COVID-19	COVID-specific	Mortality and poor outcome (ARDS, intubation or ECMO, ICU admission) as separate models; no timepoint specified	Age, sex, neutrophils, lymphocytes, platelets, CRP, creatinine	Logistic regression (LASSO)	Logistic regression

MEWS: modified early warning score; qSOFA: quick sequential (sepsis-related) organ failure assessment; REMS: rapid emergency medicine score; NEWS: national early warning score; TACTIC: therapeutic study in pre-ICU patients admitted with COVID-19; ICU: intensive care unit; AVPU: alert/responds to voice/responsive to pain/unresponsive; COVID: coronavirus disease; CVD: cardiovascular disease; CRP: C-reactive protein; LDH: lactate dehydrogenase; RALE: Radiographic Assessment of Lung Edema; COPD: chronic obstructive pulmonary disease; CT: computed tomography; ARDS: acute respiratory distress syndrome; ECMO: extracorporeal membrane oxygenation. ^#^: MEWS and REMS were evaluated among people with COVID-19 by Hu *et al.* [43], and thus were included in the present study; ^¶^: no model intercept was available so the intercepts for these models were calibrated to the validation dataset, using the model linear predictors as offset terms; ^+^: using oxygen scale 1 for all participants, except for those with target oxygen saturation ranges of 88%–92%, *e.g.* in hypercapnic respiratory failure, when scale 2 is used, as recommended [12].

### Overview of study cohort

During the study period, 521 adults were admitted with a final diagnosis of COVID-19, of whom 411 met the eligibility criteria for inclusion (supplementary figure S2). The median age of the cohort was 66 years (interquartile range (IQR) 53–79 years), and the majority were male (252 of 411; 61.3%). Table 2 shows the baseline demographics, comorbidities, laboratory results and clinical measurements of the study cohort, of which most (370 of 411; 90.0%) had PCR-confirmed SARS-CoV-2 infection (315 of 370 (85.1%) were positive on their first PCR test). A total of 180 participants (43.8%) met the endpoints of clinical deterioration and 115 participants (28.0%) met the endpoints of mortality, above the minimum requirement of 100 events recommended for external validation studies [45]. The risks of clinical deterioration and death declined with time since admission (median days to deterioration 1.4 (IQR 0.3–4.2); median days to death 6.6 (IQR 3.6–13.1); supplementary figure S3). Most of the variables required for calculating the 22 prognostic model scores were available among the vast majority of participants. However, admission lactate dehydrogenase was only available for 183 out of 411 (44.5%) and D-dimer was only measured for 153 out of 411 (37.2%), resulting in significant missingness for models requiring these variables (supplementary figure S4).

**TABLE 2:** Baseline characteristics of hospitalised adults with COVID-19 included in systematic evaluation cohort

**Variable**	**Subjects with available data**	**Overall**
**Demographics**		
Age years	411 (100)	66.0 (53.0–79.0)
Sex	411 (100)	
Female		159 (38.7)
Male		252 (61.3)
Ethnicity	390 (94.9)	
Asian		52 (13.3)
Black		56 (14.4)
White		234 (60.0)
Mixed		7 (1.8)
Other		41 (10.5)
Clinical frailty scale	411 (100)	2.0 (1.0–6.0)
**Comorbidities**		
Hypertension	411 (100)	172 (41.8)
Chronic cardiovascular disease	410 (99.8)	108 (26.3)
Chronic respiratory disease	411 (100)	99 (24.1)
Diabetes	411 (100)	105 (25.5)
Obesity^#^	411 (100)	83 (20.2)
Chronic kidney disease	410 (99.8)	40 (9.8)
**Laboratory measurements**		
C-reactive protein mg·L^−1^	403 (98.1)	96.7 (45.2–178.7)
Lymphocytes ×10^9^	410 (99.8)	0.9 (0.6–1.4)
Lactate dehydrogenase U·L^−1^	183 (44.5)	395.0 (309.0–511.0)
D-dimer ng·mL^−1^	153 (37.2)	1070.0 (640.0–2120.0)
SARS-CoV-2 PCR	411 (100)	370 (90.0)
**Physiological measurements**		
Respiratory rate breaths·min^−1^	410 (99.8)	24.0 (20.0–28.0)
Heart rate beats·min^−1^	410 (99.8)	94.0 (81.2–107.0)
Systolic blood pressure mmHg	411 (100)	131.0 (115.0–143.0)
Oxygen saturation % on air	403 (98.1)	91.0 (86.0–95.0)
**Outcome**		
Deteriorated	411 (100)	180 (43.8)
Died	411 (100)	115 (28.0)

Laboratory and physiological measurements reflect parameters at the time of hospital admission. Data are presented as n (%) or median (interquartile range). COVID-19: coronavirus disease 2019; SARS-CoV-2: severe acute respiratory syndrome coronavirus-2. ^#^: clinician-defined obesity.

### Evaluation of prognostic models for original primary outcomes

Table 3 shows discrimination and calibration metrics, where appropriate, for the 22 evaluated prognostic models in the primary multiple imputation analysis. The highest AUROCs were achieved by the NEWS2 score for prediction of deterioration over 24 h (0.78, 95% CI 0.73–0.83) and the Carr “final” model for prediction of deterioration over 14 days (0.78, 95% CI 0.74–0.82). Of the other prognostic scores currently used in routine clinical practice, CURB65 had an AUROC of 0.75 for 30-day mortality (95% CI 0.70–0.80), while qSOFA discriminated in-hospital mortality with an AUROC of 0.6 (95% CI 0.55–0.65).

**TABLE 3:** Validation metrics of prognostic scores for COVID-19, using primary multiple imputation analysis (n=411)

**Score**	**Primary outcome**	**AUROC (95% CI)**	**Calibration slope (95% CI)**	**Calibration-in-the-large (95% CI)**
**NEWS2**	Deterioration (1 day)	0.78 (0.73–0.83)		
**Ji**	Deterioration (10 days)	0.56 (0.5–0.62)		
**Carr_final**	Deterioration (14 days)	0.78 (0.74–0.82)	1.04 (0.8–1.28)	0.33 (0.11–0.55)
**Carr_threshold**	Deterioration (14 days)	0.76 (0.71–0.81)	0.85 (0.65–1.05)	−0.34 (−0.57– −0.12)
**Guo**	Deterioration (14 days)	0.67 (0.61–0.73)		
**Zhang_poor**	Deterioration (in-hospital)	0.74 (0.69–0.79)	0.33 (0.22–0.43)	0.56 (0.3–0.81)
**Galloway**	Deterioration (in-hospital)	0.72 (0.68–0.77)		
**TACTIC**	Deterioration (in-hospital)	0.7 (0.65–0.75)		
**Colombi_clinical**	Deterioration (in-hospital)	0.69 (0.63–0.74)	0.53 (0.35–0.71)	0 (−0.23–0.23)
**Huang**	Deterioration (in-hospital)	0.67 (0.61–0.73)	0.18 (0.1–0.26)	−4.26 (−4.61– −3.91)
**Shi**	Deterioration (in-hospital)	0.61 (0.56–0.66)		
**MEWS**	Deterioration (in-hospital)	0.6 (0.54–0.65)		
**Lu**	Mortality (12 days)	0.72 (0.67–0.76)		
**CURB65**	Mortality (30 days)	0.75 (0.7–0.8)		
**BelloChavolla**	Mortality (30 days)	0.66 (0.6–0.72)		
**REMS**	Mortality (in-hospital)	0.76 (0.71–0.81)		
**Xie**	Mortality (in-hospital)	0.76 (0.69–0.82)	0.83 (0.51–1.15)	0.41 (0.16–0.66)
**Hu**	Mortality (in-hospital)	0.74 (0.68–0.79)	0.33 (0.2–0.45)	−1.07 (−1.37– −0.77)
**Caramelo**	Mortality (in-hospital)	0.71 (0.66–0.76)	0.53 (0.36–0.69)	0 (−0.25–0.25)
**Zhang_death**	Mortality (in-hospital)	0.7 (0.65–0.76)	0.29 (0.19–0.4)	0.89 (0.6–1.19)
**qSOFA**	Mortality (in-hospital)	0.6 (0.55–0.65)		
**Yan**	Mortality (in-hospital)	0.58 (0.49–0.67)		

For each model, performance is evaluated for its original intended outcome, shown in “Primary outcome” column. AUROC: area under the receiver operating characteristic curve.

**FIGURE 1:**
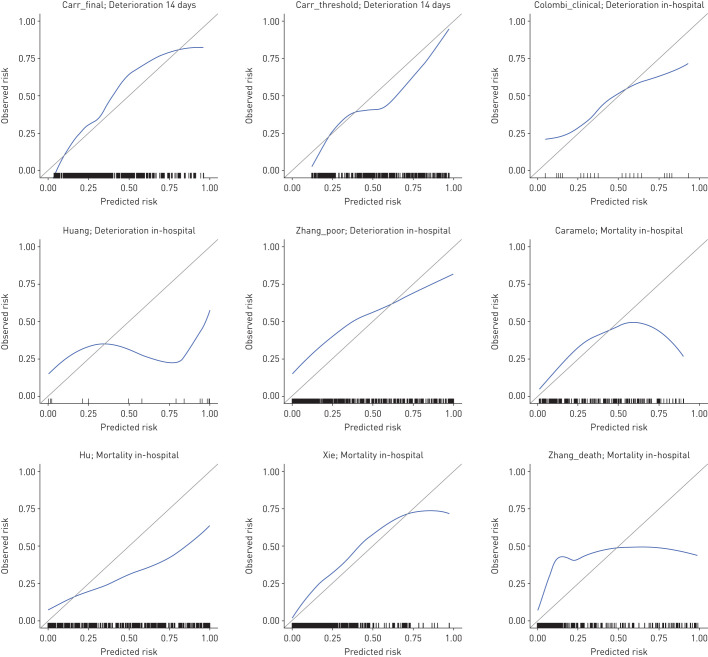
Calibration plots for prognostic models estimating outcome probabilities. For each plot, the blue line represents a LOESS-smoothed calibration curve from the stacked multiple imputed datasets and rug plots indicate the distribution of data points. No model intercept was available for the Caramelo or Colombi “clinical” models; the intercepts for these models were calibrated to the validation dataset using the model linear predictors as offset terms. The primary outcome of interest for each model is shown in the plot sub-heading.

For all models that provide probability scores for either deterioration or mortality, calibration appeared visually poor with evidence of overfitting and either systematic overestimation or underestimation of risk (figure 1). Supplementary figure S5 shows associations between prognostic models with points-based scores and actual risk. In addition to demonstrating reasonable discrimination, the NEWS2 and CURB65 models demonstrated approximately linear associations between scores and actual probability of deterioration at 24 h and mortality at 30 days, respectively.

### Time-dependent discrimination of candidate models and *a priori* univariable predictors for standardised outcomes

Next, we sought to compare the discrimination of these models for both clinical deterioration and mortality across the range of time horizons, benchmarked against preselected univariable predictors associated with adverse outcomes in COVID-19 [8, 19]. We recalculated time-dependent AUROCs for each of these outcomes, stratified by time horizon to the outcome (supplementary figures S6 and S7). These analyses showed that AUROCs generally declined with increasing time horizons. Admission peripheral oxygen saturation on room air was the strongest predictor of in-hospital deterioration (AUROC 0.76, 95% CI 0.71–0.81), while age was the strongest predictor of in-hospital mortality (AUROC 0.76, 95% CI 0.71–0.81).

### Decision curve analyses to assess clinical utility

**FIGURE 2:**
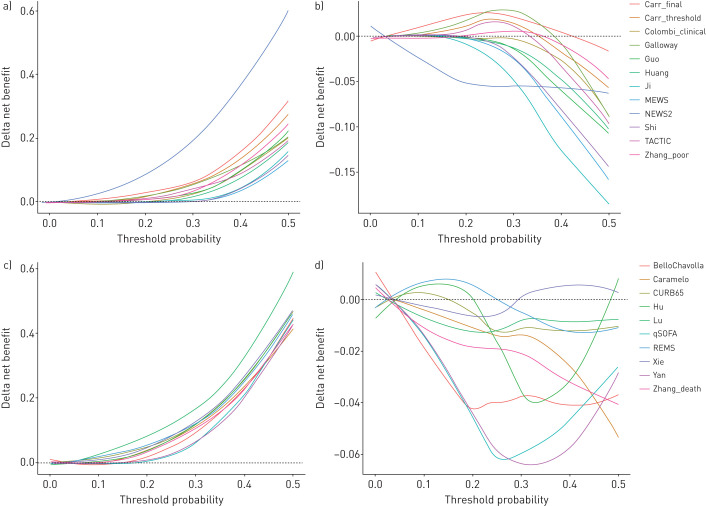
Decision curve analysis showing delta net benefit of each candidate model, compared to treating all patients and best univariable predictors. a) Deterioration models *versus* treat all; b) deterioration models *versus* peripheral oxygen saturation (*S*_pO_2__) on air alone; c) mortality models *versus* treat all; d) mortality models *versus* age alone. For each analysis, the endpoint is the original intended outcome and time horizon for the index model. Each candidate model and univariable predictor was calibrated to the validation data during analysis to enable fair, head-to-head comparisons. Delta net benefit is calculated as net benefit when using the index model minus net benefit when 1) treating all patients and 2) using the most discriminating univariable predictor. The most discriminating univariable predictor is admission *S*_pO_2__ on room air for deterioration models and patient age for mortality models. Delta net benefit is shown with LOESS-smoothing. Black dashed line indicates threshold above which index model has greater net benefit than the comparator. Individual decision curves for each candidate model are shown in supplementary figure S8.

We compared net benefit for each prognostic model (for its original intended endpoint) to the strategies of treating all patients, treating no patients and using the most discriminating univariable predictor for either deterioration (*i.e.* oxygen saturation on air) or mortality (*i.e.* patient age) to stratify treatment (supplementary figure S8). Although all prognostic models showed greater net benefit than treating all patients at the higher range of threshold probabilities, none of these models demonstrated consistently greater net benefit than the most discriminating univariable predictor, across the range of threshold probabilities (figure 2).

### Sensitivity analyses

Recalculation of model discrimination and calibration metrics for predicting the original intended endpoint using 1) a complete case analysis, 2) excluding patients without PCR-confirmed SARS-CoV-2 infection and 3) excluding patients who met the clinical deterioration outcome within 4 h of arrival to hospital revealed similar results to the primary multiple imputation approach, though discrimination was noted to be lower overall when excluding early events (supplementary table S2). Visual examination of associations between the most discriminating univariable predictors and log odds of deterioration or death using restricted cubic splines showed no evidence of non-linear associations (supplementary figure S9). Finally, internal validation using bootstrapping showed near zero optimism for discrimination and calibration parameters for the univariable models (supplementary table S3).

## Discussion

In this observational cohort study of consecutive adults hospitalised with COVID-19, we systematically evaluated the performance of 22 prognostic models for COVID-19. These included models developed specifically for COVID-19, along with existing scores in routine clinical use prior to the pandemic. For prediction of clinical deterioration or mortality, AUROCs ranged from 0.56–0.78. NEWS2 performed reasonably well for prediction of deterioration over a 24-h interval, achieving an AUROC of 0.78, while the Carr “final” model [30] also had an AUROC of 0.78, but tended to systematically underestimate risk. All COVID-specific models that derived an outcome probability of either deterioration or mortality showed poor calibration. We found that peripheral oxygen saturation on room air (AUROC 0.76) and patient age (AUROC 0.76) were the most discriminating single variables for prediction of in-hospital deterioration and mortality respectively. These predictors have the added advantage that they are immediately available at the point of presentation to hospital. In decision curve analysis, which is dependent upon both model discrimination and calibration, no prognostic model demonstrated clinical utility consistently greater than using these univariable predictors to inform decision-making.

While previous studies have largely focused on novel model discovery, or evaluating a limited number of existing models, this is the first study to our knowledge to evaluate systematically identified candidate prognostic models for COVID-19. We used a comprehensive living systematic review [8] to identify eligible models and sought to reconstruct each model as per the original authors’ description. We then evaluated performance against its intended outcome and time horizon, wherever possible, using recommended methods of external validation incorporating assessments of discrimination, calibration and net benefit [17]. We used a robust approach of electronic health record data capture, supported by manual curation, to ensure a high-quality dataset, and inclusion of unselected and consecutive COVID-19 cases that met our eligibility criteria. We used robust outcome measures of mortality and clinical deterioration, aligning with the WHO Clinical Progression Scale [16].

A weakness of the current study is that it is based on retrospective data from a single centre, and therefore cannot assess between-setting heterogeneity in model performance. Second, owing to the limitations of routinely collected data, predictor variables were available for varying numbers of participants for each model, with a large proportion of missingness for models requiring lactate dehydrogenase and D-dimer measurements. We therefore performed multiple imputation, in keeping with recommendations for development and validation of multivariable prediction models, in our primary analyses [46]. Findings were similar in the complete case sensitivity analysis, thus supporting the robustness of our results. Future studies would benefit from standardising data capture and laboratory measurements prospectively to minimise predictor missingness. Third, a number of models could not be reconstructed in our data. For some models, this was due the absence of predictors in our dataset, such as those requiring computed tomography imaging, because this is not currently routinely recommended for patients with suspected or confirmed COVID-19 [15]. We were also not able to include models for which the parameters were not publicly available. This underscores the need for strict adherence to reporting standards in multivariable prediction models [13]. Finally, we used admission data only as predictors in this study, because most prognostic scores are intended to predict outcomes at the point of hospital admission. We note, however, that some scores are designed for dynamic inpatient monitoring, with NEWS2 showing reasonable discrimination for deterioration over a 24-h interval, as originally intended [44]. Future studies may integrate serial data to examine model performance when using such dynamic measurements.

Despite the vast global interest in the pursuit of prognostic models for COVID-19, our findings show that none of the COVID-19-specific models evaluated in this study can currently be recommended for routine clinical use. In addition, while some of the evaluated models that are not specific to COVID-19 are routinely used and may be of value among inpatients [12, 44], people with suspected infection [10] or community-acquired pneumonia [11], none showed greater clinical utility than the strongest univariable predictors among patients with COVID-19. Our data show that admission oxygen saturation on room air is a strong predictor of clinical deterioration and suggest that it should be evaluated in future studies to stratify inpatient management and for remote community monitoring. We note that all novel prognostic models for COVID-19 assessed in the current study were derived from single-centre data. Future studies may seek to pool data from multiple centres to robustly evaluate the performance of existing and newly emerging models across heterogeneous populations, and develop and validate novel prognostic models, through individual participant data meta-analysis [47]. Such an approach would allow assessments of between-study heterogeneity and the likely generalisability of candidate models. It is also imperative that discovery populations are representative of target populations for model implementation, with inclusion of unselected cohorts. Moreover, we strongly advocate for transparent reporting in keeping with TRIPOD standards (including modelling approaches, all model coefficients and standard errors) along with standardisation of outcomes and time horizons, to facilitate ongoing systematic evaluations of model performance and clinical utility [13].

We conclude that baseline oxygen saturation on room air and patient age are strong predictors of deterioration and mortality, respectively. None of the prognostic models evaluated in this study offer incremental value for patient stratification to these univariable predictors when using admission data. Therefore, none of the evaluated prognostic models for COVID-19 can be recommended for routine clinical implementation. Future studies seeking to develop prognostic models for COVID-19 should consider integrating multi-centre data to increase generalisability of findings, and should ensure benchmarking against existing models and simpler univariable predictors.

## Supplementary material

10.1183/13993003.03498-2020.Supp1 **Please note:** supplementary material is not edited by the Editorial Office, and is uploaded as it has been supplied by the author.
Supplementary material ERJ-03498-2020.Supplement

## Shareable PDF

10.1183/13993003.03498-2020.Shareable1 This one-page PDF can be shared freely online.
Shareable PDF ERJ-03498-2020.Shareable
