# Supplementary material for: Systematic evaluation and external validation of 22 prognostic models among hospitalised adults with COVID-19: an observational cohort study
Source: Eur Respir J. 2020 Dec 24;56(6):2003498. doi: 10.1183/13993003.03498-2020 (PMC7518075; doi:10.1183/13993003.03498-2020)
Supplement: Supplementary file 1 [file ERJ-03498-2020.Supplement.pdf]

Systematic evaluation and external validation of 22 prognostic models among hospitalised adults with COVID-19: An observational cohort study

Supplementary Material

Supplementary Table 1: Characteristics of candidate prognostic models for COVID-19 not included in current external validation study.

All candidate models included in a living systematic review were considered at high risk of bias[1]. ARDS = acute respiratory distress syndrome; ICU = intensive care unit; CT = computed tomography.

**Authors**	**Pre-existing or COVID-specific?**	**Model outcome**	**Reason for exclusion**
Bai et al[2]	COVID-specific	Deterioration	CT imaging required
Barda et al[3]	COVID-specific	Mortality	Multiple predictors not available in validation data
Chassagnon et al[4]	COVID-specific	Death or invasive mechanical ventilation	CT imaging required
Das et al[5]	COVID-specific	Mortality	Province required - not generalisable, and not reproducible
Gong et al[6]	COVID-specific	Risk of severe disease	Model parameters not publicly available
Jiang et al[7]	COVID-specific	Development of ARDS by Berlin criteria	Model parameters not publicly available
Levy et al[8]	COVID-specific	Mortality	Emergency severity index not available in validation data
Liang et al[9]	COVID-specific	Deterioration	Symptom data not available in validation data
Liu et al[10]	COVID-specific	Severity	T lymphocyte subsets not available in validation data
McRae et al[11]	COVID-specific	Mortality	Pro-calcitonin and myoglobin not available; exact model coefficients also not provided
Pourhomayoun et al[12]	COVID-specific	Mortality	Model parameters not publicly available
Qi et al[13]	COVID-specific	Length of hospital stay	CT imaging required
Sarkar et al[14]	COVID-specific	Mortality	Not generalisable outside Wuhan and model not reproducible
Singh et al[15]	Pre-existing (EPIC deterioration index)	ICU admission, mechanical ventilation or death	Model parameters not publicly available
Vaid et al[16]	COVID-specific	Mortality and 'critical events'	Model parameters not publicly available
Vazquez et al[17]	COVID-specific	Mortality (in-hospital)	Intended for ICU admissions only
Yuan et al[18]	COVID-specific	Mortality	CT imaging required
Zeng et al[19]	COVID-specific	Progression to severe disease	CT imaging required and no reproducible model

Supplementary Tables 2a-c: Validation metrics of prognostic scores for COVID-19, using (a) complete case sensitivity analysis (n=411); (b) excluding patients without PCR-confirmed SARS-CoV-2 infection; and (c) excluding patients who met the clinical deterioration outcome within 4 hours of arrival to hospital..

For each model, performance is evaluated for an approximation of its original intended outcome, shown in 'Primary outcome' column. AUROC = area under the receiver operating characteristic curve; CI = confidence interval.

(a) Complete case analysis

Score	Primary outcome	n	AUROC (95% CI)	Calibration slope (95% CI)	Calibration in the large (95% CI)
NEWS2	Deterioration (1 day)	404	0.78 (0.73 - 0.83)		
Ji	Deterioration (10 days)	183	0.61 (0.53 - 0.69)		
Carr_final	Deterioration (14 days)	381	0.75 (0.71 - 0.8)	0.93 (0.7 - 1.18)	0.46 (0.23 - 0.69)
Carr_threshold	Deterioration (14 days)	381	0.74 (0.69 - 0.79)	0.78 (0.59 - 0.99)	-0.29 (-0.52 - -0.05)
Guo	Deterioration (14 days)	153	0.7 (0.62 - 0.78)		
Zhang_poor	Deterioration (in-hospital)	400	0.74 (0.69 - 0.78)	0.31 (0.21 - 0.42)	0.59 (0.32 - 0.83)
Colombi_clinical	Deterioration (in-hospital)	182	0.72 (0.64 - 0.79)	0.64 (0.38 - 0.93)	0 (-0.34 - 0.33)
Galloway	Deterioration (in-hospital)	351	0.7 (0.65 - 0.75)		
Huang	Deterioration (in-hospital)	182	0.7 (0.63 - 0.78)	0.27 (0.16 - 0.38)	-5.09 (-5.54 - -4.65)
TACTIC	Deterioration (in-hospital)	366	0.68 (0.63 - 0.73)		
Shi	Deterioration (in-hospital)	411	0.61 (0.56 - 0.66)		
MEWS	Deterioration (in-hospital)	405	0.59 (0.54 - 0.65)		
Lu	Mortality (12 days)	403	0.71 (0.67 - 0.76)		
CURB65	Mortality (30 days)	374	0.75 (0.7 - 0.8)		
BelloChavolla	Mortality (30 days)	385	0.66 (0.6 - 0.72)		
Xie	Mortality (in-hospital)	183	0.78 (0.7 - 0.86)	0.91 (0.55 - 1.31)	0.03 (-0.37 - 0.41)
REMS	Mortality (in-hospital)	404	0.76 (0.71 - 0.81)		
Hu	Mortality (in-hospital)	153	0.75 (0.66 - 0.84)	0.38 (0.21 - 0.58)	-1.61 (-2.11 - -1.14)
Caramelo	Mortality (in-hospital)	408	0.71 (0.66 - 0.77)	0.53 (0.36 - 0.7)	0 (-0.26 - 0.25)
Zhang_death	Mortality (in-hospital)	400	0.7 (0.64 - 0.76)	0.28 (0.18 - 0.39)	0.92 (0.63 - 1.21)
qSOFA	Mortality (in-hospital)	405	0.6 (0.54 - 0.65)		
Yan	Mortality (in-hospital)	182	0.6 (0.52 - 0.68)		
Age	Mortality (in-hospital)	411	0.76 (0.71 - 0.81)		
SpO2 on air	Deterioration (in-hospital)	403	0.76 (0.71 - 0.81)		

(b) Restriction to PCR-confirmed cases (n=370)

Score	Primary outcome	AUROC (95% CI)	Calibration slope (95% CI)	Calibration in the large (95% CI)
NEWS2	Deterioration (1 day)	0.79 (0.74 - 0.84)		
Ji	Deterioration (10 days)	0.58 (0.52 - 0.64)		
Carr_final	Deterioration (14 days)	0.78 (0.73 - 0.83)	1.02 (0.77 - 1.27)	0.38 (0.14 - 0.61)
Carr_threshold	Deterioration (14 days)	0.76 (0.71 - 0.81)	0.83 (0.62 - 1.04)	-0.3 (-0.54 - -0.06)
Guo	Deterioration (14 days)	0.68 (0.62 - 0.74)		
Zhang_poor	Deterioration (in-hospital)	0.74 (0.69 - 0.79)	0.3 (0.19 - 0.41)	0.64 (0.38 - 0.91)
Galloway	Deterioration (in-hospital)	0.73 (0.68 - 0.78)		
TACTIC	Deterioration (in-hospital)	0.71 (0.65 - 0.76)		
Colombi_clinical	Deterioration (in-hospital)	0.68 (0.63 - 0.74)	0.52 (0.33 - 0.71)	0.02 (-0.23 - 0.26)
Huang	Deterioration (in-hospital)	0.67 (0.61 - 0.73)	0.18 (0.1 - 0.26)	-4.2 (-4.58 - -3.81)
Shi	Deterioration (in-hospital)	0.61 (0.56 - 0.67)		
MEWS	Deterioration (in-hospital)	0.6 (0.55 - 0.66)		
Lu	Mortality (12 days)	0.73 (0.69 - 0.78)		
CURB65	Mortality (30 days)	0.74 (0.69 - 0.79)		
BelloChavolla	Mortality (30 days)	0.66 (0.6 - 0.72)		
REMS	Mortality (in-hospital)	0.76 (0.71 - 0.81)		
Xie	Mortality (in-hospital)	0.75 (0.68 - 0.82)	0.78 (0.46 - 1.1)	0.46 (0.2 - 0.71)
Hu	Mortality (in-hospital)	0.73 (0.67 - 0.79)	0.33 (0.2 - 0.46)	-0.99 (-1.3 - -0.67)
Zhang_death	Mortality (in-hospital)	0.71 (0.65 - 0.76)	0.3 (0.19 - 0.41)	0.99 (0.69 - 1.29)
Caramelo	Mortality (in-hospital)	0.7 (0.65 - 0.76)	0.5 (0.32 - 0.67)	0.02 (-0.24 - 0.28)
qSOFA	Mortality (in-hospital)	0.6 (0.55 - 0.66)		
Yan	Mortality (in-hospital)	0.58 (0.49 - 0.67)		
SpO2 on air	Deterioration (in-hospital)	0.76 (0.71 - 0.81)		
Age	Mortality (in-hospital)	0.75 (0.7 - 0.81)		

(c) Excluding deterioration events <4 hours from admission (n=371)

Score	Primary outcome	AUROC (95% CI)	Calibration slope (95% CI)	Calibration in the large (95% CI)
NEWS2	Deterioration (1 day)	0.74 (0.67 - 0.81)		
Ji	Deterioration (10 days)	0.55 (0.49 - 0.62)		
Carr_final	Deterioration (14 days)	0.74 (0.69 - 0.79)	0.87 (0.62 - 1.12)	0.14 (-0.1 - 0.37)
Carr_threshold	Deterioration (14 days)	0.73 (0.67 - 0.78)	0.73 (0.52 - 0.94)	-0.54 (-0.78 - -0.29)
Guo	Deterioration (14 days)	0.65 (0.58 - 0.71)		
Zhang_poor	Deterioration (in-hospital)	0.72 (0.67 - 0.78)	0.39 (0.27 - 0.51)	0.3 (0.03 - 0.58)
Galloway	Deterioration (in-hospital)	0.69 (0.64 - 0.75)		
Colombi_clinical	Deterioration (in-hospital)	0.68 (0.62 - 0.74)	0.48 (0.29 - 0.66)	-0.25 (-0.5 - -0.01)
TACTIC	Deterioration (in-hospital)	0.66 (0.6 - 0.71)		
Huang	Deterioration (in-hospital)	0.63 (0.56 - 0.71)	0.14 (0.06 - 0.23)	-4.49 (-4.84 - -4.15)
Shi	Deterioration (in-hospital)	0.59 (0.54 - 0.65)		
MEWS	Deterioration (in-hospital)	0.56 (0.5 - 0.62)		
Lu	Mortality (12 days)	0.73 (0.69 - 0.78)		
CURB65	Mortality (30 days)	0.74 (0.69 - 0.8)		
BelloChavolla	Mortality (30 days)	0.65 (0.59 - 0.72)		
REMS	Mortality (in-hospital)	0.76 (0.71 - 0.81)		
Xie	Mortality (in-hospital)	0.75 (0.68 - 0.82)	0.83 (0.52 - 1.13)	0.32 (0.05 - 0.59)
Hu	Mortality (in-hospital)	0.73 (0.67 - 0.79)	0.34 (0.21 - 0.48)	-1.07 (-1.38 - -0.75)
Caramelo	Mortality (in-hospital)	0.71 (0.65 - 0.77)	0.51 (0.33 - 0.69)	-0.18 (-0.46 - 0.09)
Zhang_death	Mortality (in-hospital)	0.69 (0.63 - 0.75)	0.29 (0.17 - 0.4)	0.88 (0.57 - 1.19)
qSOFA	Mortality (in-hospital)	0.59 (0.53 - 0.66)		
Yan	Mortality (in-hospital)	0.57 (0.48 - 0.66)		
Age	Mortality (in-hospital)	0.77 (0.71 - 0.82)		
SpO2 on air	Deterioration (in-hospital)	0.7 (0.64 - 0.76)		

Supplementary Table 3: Optimism estimates for most discriminating univariable predictors of clinical deterioration and mortality

Optimism is calculated using bootstrapping with 1,000 iterations. AUROC = area under the receiver operating characteristic curve; CI = confidence interval. Dxy = Somers' Delta, which is a measure of agreement between pairs of ordinal variables, ranging from -1 (no agreement) to +1 (complete agreement).

**Predictor**	**Outcome**	**AUROC (95% CI)**	**Optimism**	
			Dxy	*Slope*
Age	Mortality (in-hospital)	0.76 (0.71 - 0.81)	0.000	-0.009
Oxygen saturation (room air)	Deterioration (in-hospital)	0.76 (0.71 - 0.81)	-0.001	-0.011

Supplementary Figure 1: Flowchart showing prognostic models included in systematic evaluation.

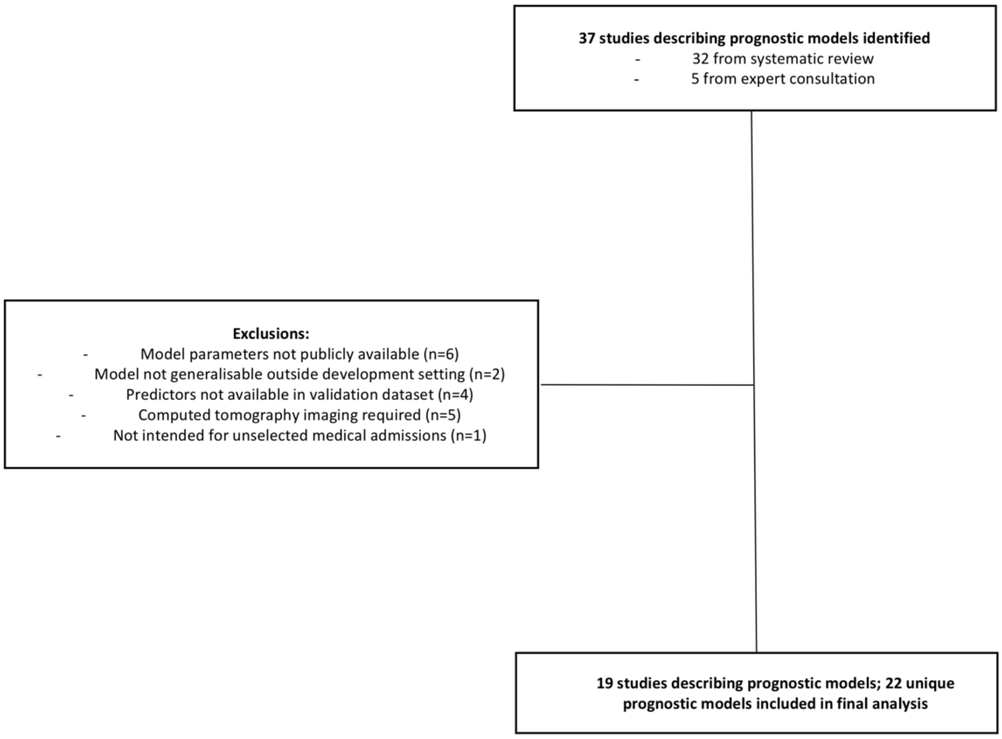

Supplementary Figure 2: Flowchart showing study participants included in analysis.

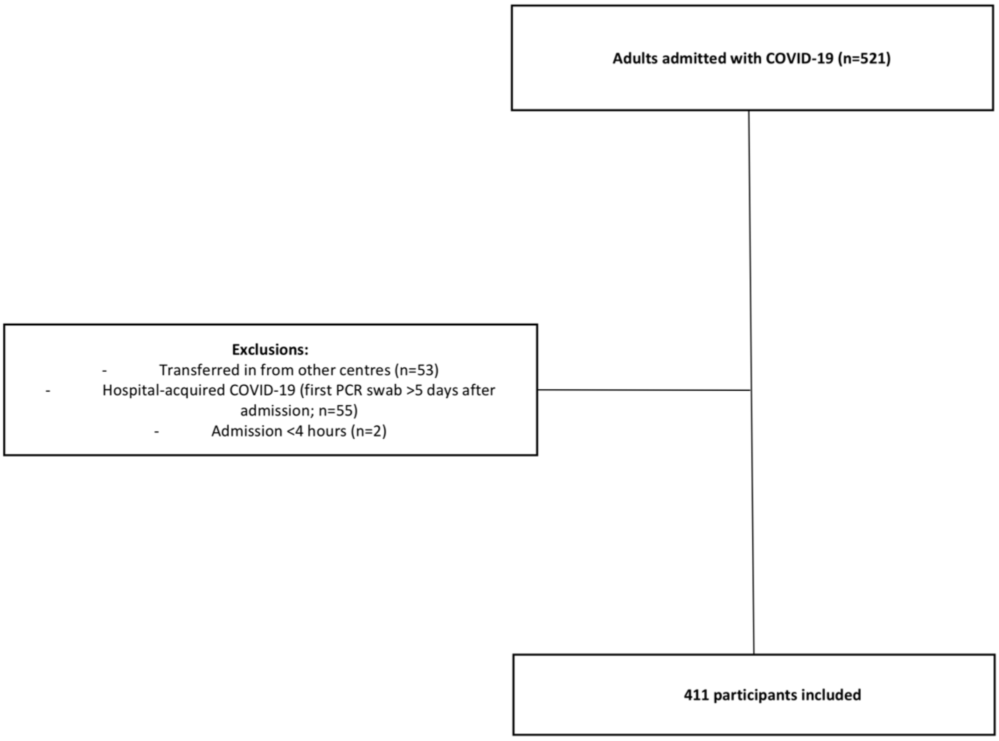

Supplementary Figure 3: Timing of clinical deterioration and death following hospital admission among patients with COVID-19

Shown as Kaplan-Meier plots and histograms. Days to deterioration histogram reflects the first time that the endpoint was met, with the criteria for meeting the endpoint indicated by colour. CPAP = continuous positive airway pressure; HFNO = high flow nasal cannula oxygen; IMV = invasive mechanical ventilation.

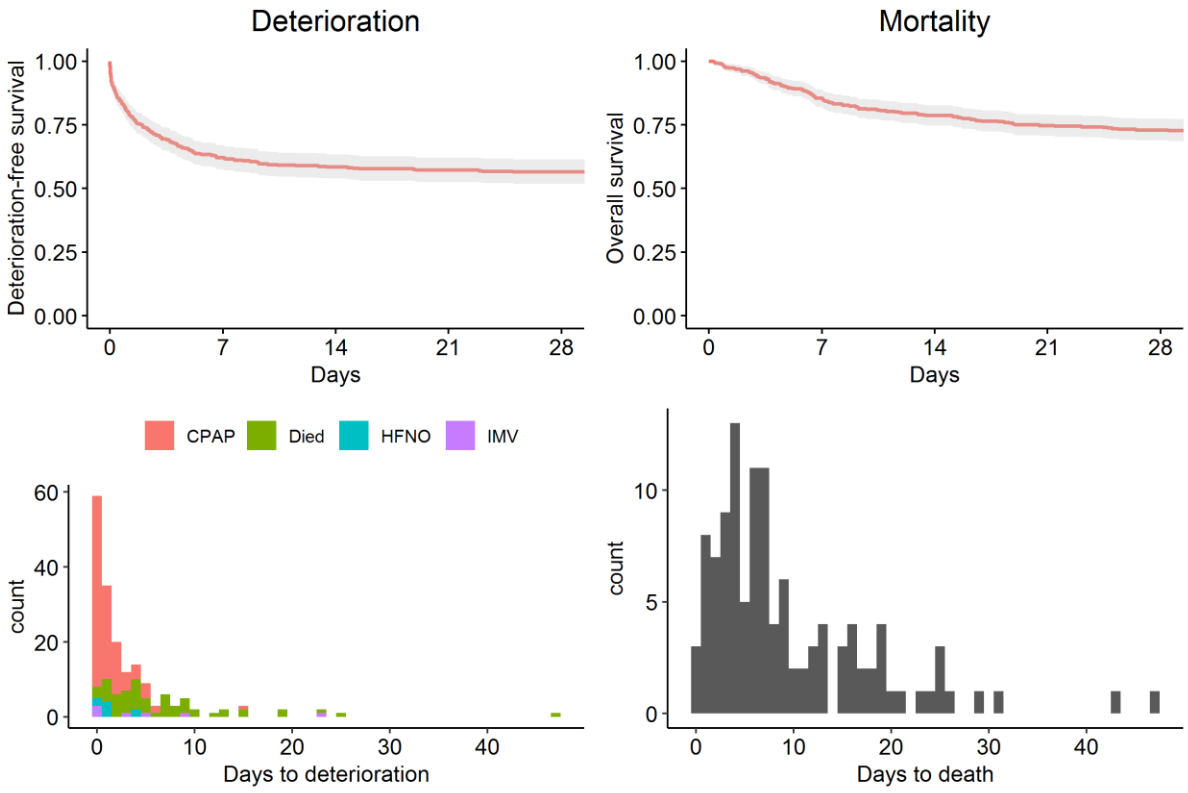

Supplementary Figure 4: Missingness of candidate prognostic models.

Shown as column-wise percentage missing, stratified by (a) composite outcome of clinical deterioration; and (b) mortality during hospital admission.

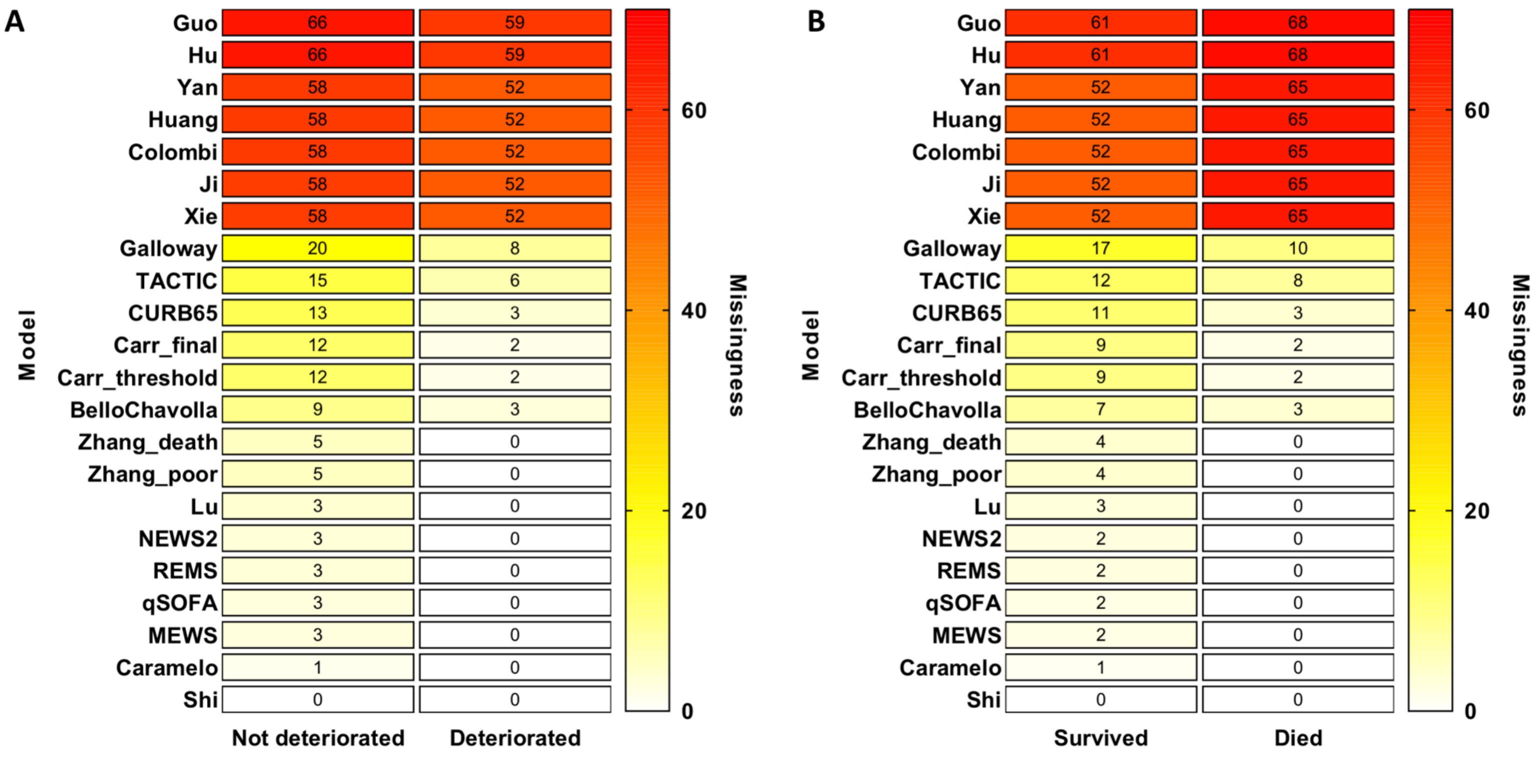

Supplementary Figure 5: Plots showing risk scores vs observed prevalence of outcomes for points-based prognostic scores.

The primary outcome of interest for each model is shown in the plot sub-heading. Individual predictions for each prognostic model were averaged across imputations for each participant in the dataset in order to generate these pooled plots.

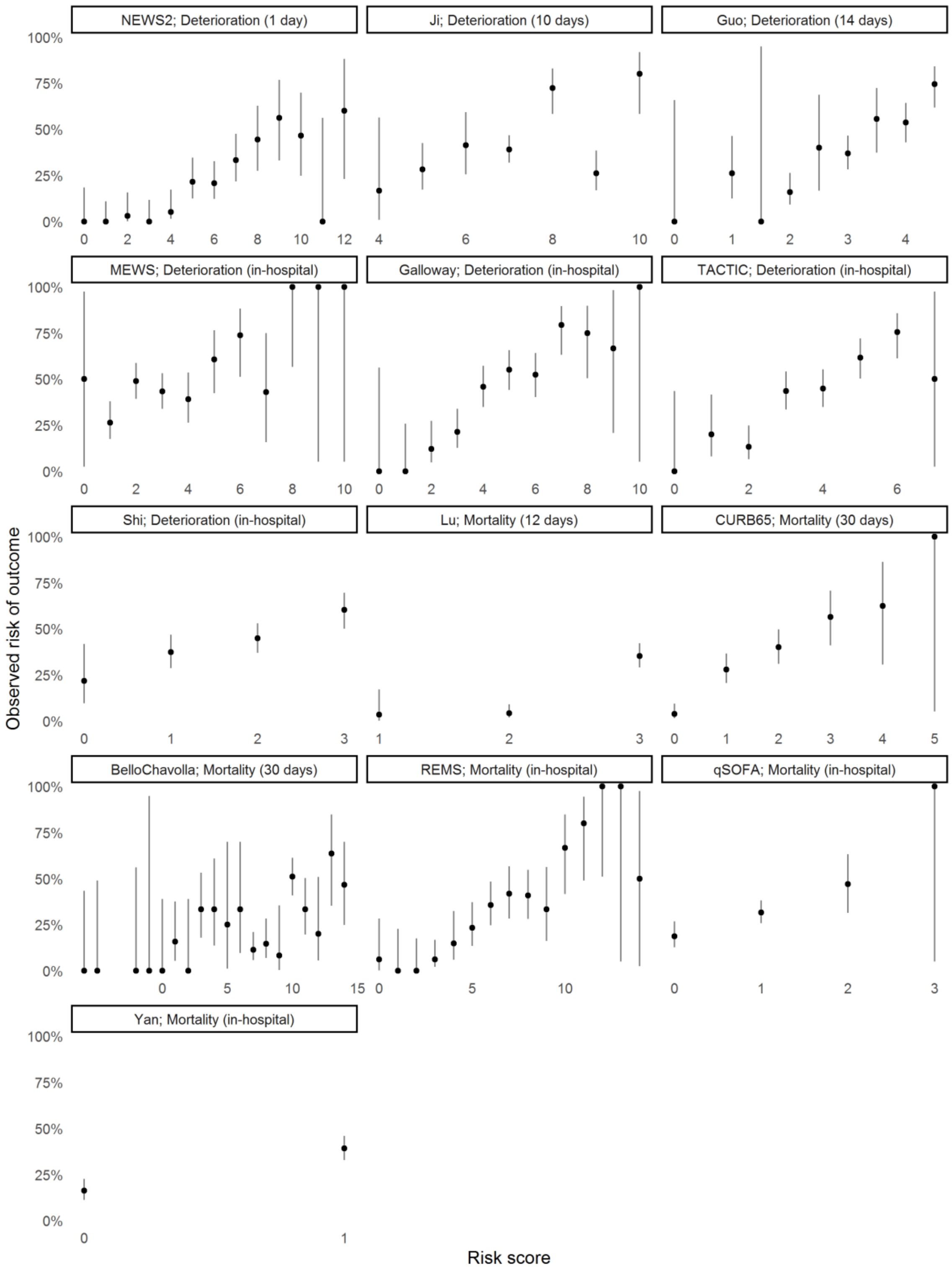
NEWS2; Deterioration (1 day)
Ji; Deterioration (10 days)
Guo; Deterioration (14 days)
MEWS; Deterioration (in-hospital)
Galloway; Deterioration (in-hospital)
TACTIC; Deterioration (in-hospital)
Shi; Deterioration (in-hospital)
Lu; Mortality (12 days)
CURB65; Mortality (30 days)
BelloChavolla; Mortality (30 days)
REMS; Mortality (in-hospital)
qSOFA; Mortality (in-hospital)
Yan; Mortality (in-hospital)
Observed risk of outcome
Risk score

Supplementary Figure 6: Heat map showing time-dependent receiver operating characteristic areas under the curve for each prognostic score to predict (a) deterioration or (b) mortality,

For each model, discrimination is stratified by interval from hospital admission to the outcome event. The original intended primary outcome for the model is shown in brackets in the y-axis labels. AUROC = area under the receiver operating characteristic curve.

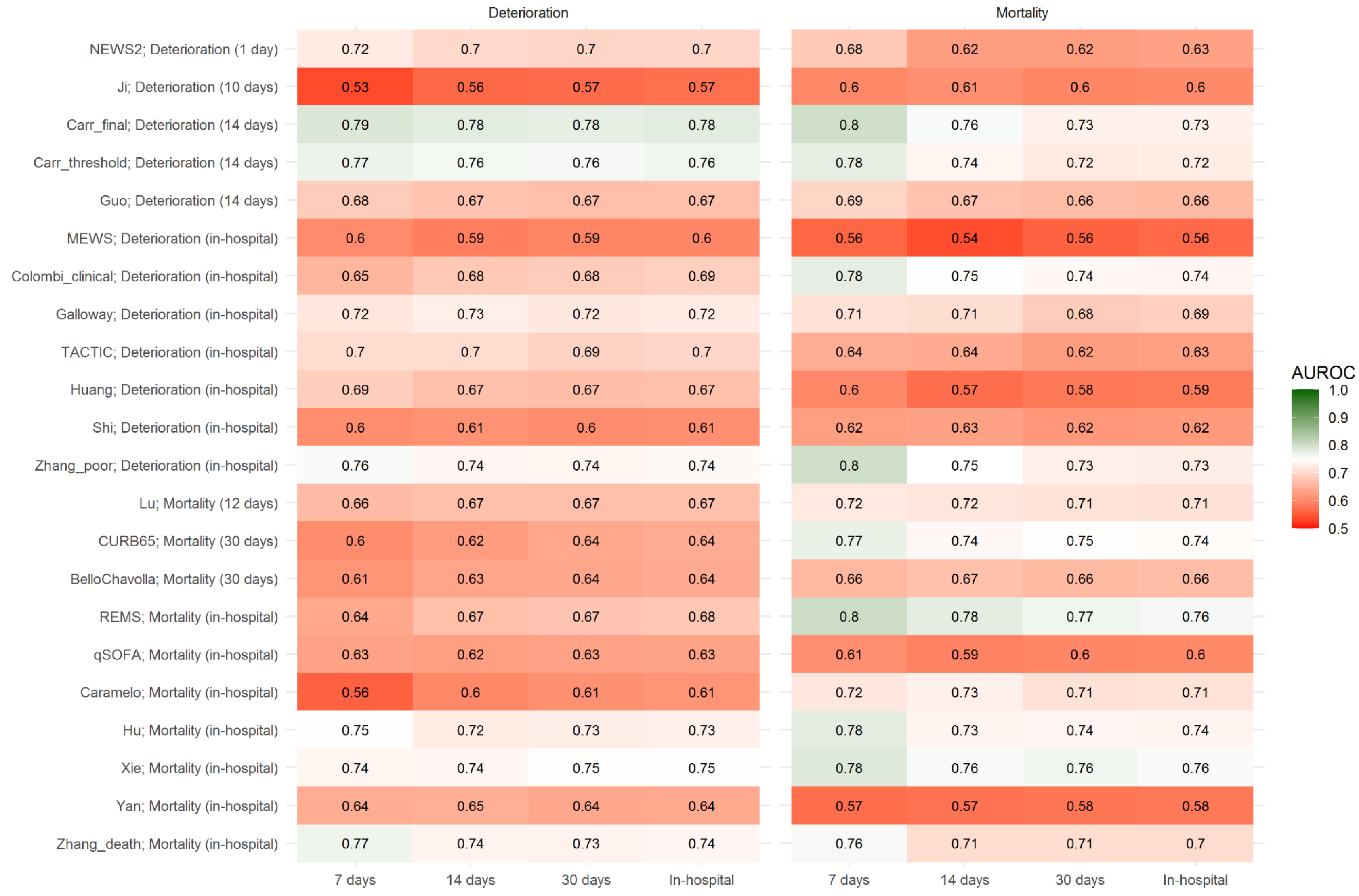
Deterioration
Mortality
NEWS2; Deterioration (1 day) 0.72 0.7 0.7 0.7 0.68 0.62 0.62 0.63
Ji; Deterioration (10 days) 0.53 0.56 0.57 0.57 0.6 0.61 0.6 0.6
Carr_final; Deterioration (14 days) 0.79 0.78 0.78 0.78 0.8 0.76 0.73 0.73
Carr_threshold; Deterioration (14 days) 0.77 0.76 0.76 0.76 0.78 0.74 0.72 0.72
Guo; Deterioration (14 days) 0.68 0.67 0.67 0.67 0.69 0.67 0.66 0.66
MEWS; Deterioration (in-hospital) 0.6 0.59 0.59 0.6 0.56 0.54 0.56 0.56
Colombi_clinical; Deterioration (in-hospital) 0.65 0.68 0.68 0.69 0.78 0.75 0.74 0.74
Galloway; Deterioration (in-hospital) 0.72 0.73 0.72 0.72 0.71 0.71 0.68 0.69
TACTIC; Deterioration (in-hospital) 0.7 0.7 0.69 0.7 0.64 0.64 0.62 0.63
Huang; Deterioration (in-hospital) 0.69 0.67 0.67 0.67 0.6 0.57 0.58 0.59
Shi; Deterioration (in-hospital) 0.6 0.61 0.6 0.61 0.62 0.63 0.62 0.62
Zhang_poor; Deterioration (in-hospital) 0.76 0.74 0.74 0.74 0.8 0.75 0.73 0.73
Lu; Mortality (12 days) 0.66 0.67 0.67 0.67 0.72 0.72 0.71 0.71
CURB65; Mortality (30 days) 0.6 0.62 0.64 0.64 0.77 0.74 0.75 0.74
BelloChavolla; Mortality (30 days) 0.61 0.63 0.64 0.64 0.66 0.67 0.66 0.66
REMS; Mortality (in-hospital) 0.64 0.67 0.67 0.68 0.8 0.78 0.77 0.76
qSOFA; Mortality (in-hospital) 0.63 0.62 0.63 0.63 0.61 0.59 0.6 0.6
Caramelo; Mortality (in-hospital) 0.56 0.6 0.61 0.61 0.72 0.73 0.71 0.71
Hu; Mortality (in-hospital) 0.75 0.72 0.73 0.73 0.78 0.73 0.74 0.74
Xie; Mortality (in-hospital) 0.74 0.74 0.75 0.75 0.78 0.76 0.76 0.76
Yan; Mortality (in-hospital) 0.64 0.65 0.64 0.64 0.57 0.57 0.58 0.58
Zhang_death; Mortality (in-hospital) 0.77 0.74 0.73 0.74 0.76 0.71 0.71 0.7
7 days
14 days
30 days
In-hospital
7 days
14 days
30 days
In-hospital
AUROC
1.0
0.9
0.8
0.7
0.6
0.5

Supplementary Figure 7: Heat map showing time-dependent receiver operating characteristic areas under the curve for a priori clinical predictors of interest in univariable analyses to predict (a) clinical deterioration or (b) mortality

For each predictor, discrimination is stratified by interval from hospital admission to the outcome event. AUROC = area under the receiver operating characteristic curve.

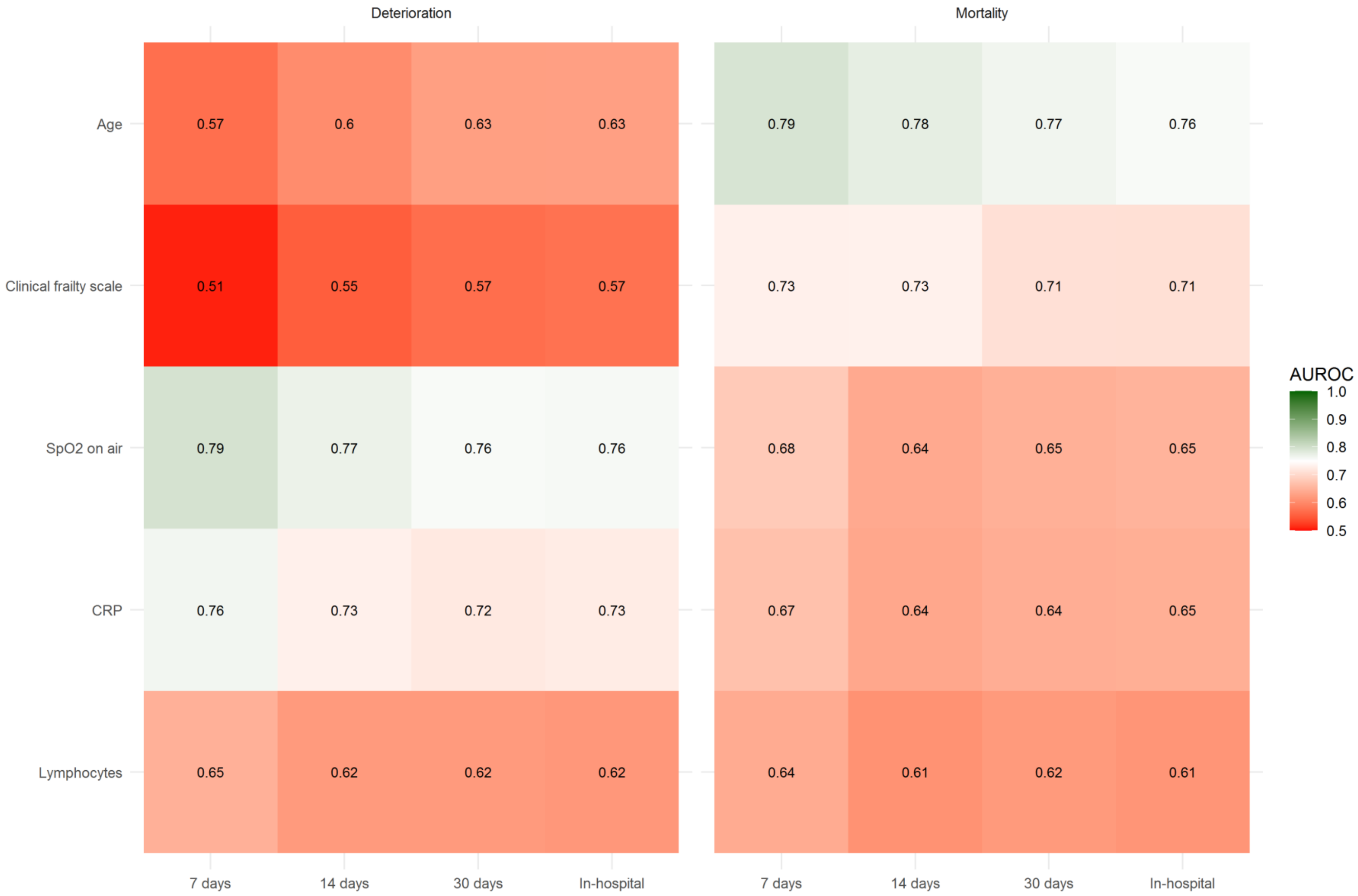
Deterioration
Mortality
Age
Clinical frailty scale
SpO2 on air
CRP
Lymphocytes
0.57
0.6
0.63
0.63
0.79
0.78
0.77
0.76
0.51
0.55
0.57
0.57
0.73
0.73
0.71
0.71
0.79
0.77
0.76
0.76
0.68
0.64
0.65
0.65
0.76
0.73
0.72
0.73
0.67
0.64
0.64
0.65
0.65
0.62
0.62
0.62
0.64
0.61
0.62
0.61
7 days
14 days
30 days
In-hospital
7 days
14 days
30 days
In-hospital
AUROC
1.0
0.9
0.8
0.7
0.6
0.5

Supplementary Figure 8: Decision curve analysis comparing net benefit of each candidate model for (A) clinical deterioration; and (B) mortality.

For each analysis, the endpoint is the original intended endpoint for the index model (purple line; endpoint shown in plot subheading). Comparisons are made to the strategies of treating all patients (red); treating no patients (blue); and offering treatment based on the most discriminating univariable predictor (green; admission oxygen saturation on room air for deterioration models (A); patient age for mortality models (B)). Each candidate model and univariable predictor was recalibrated to the validation data during analysis to enable fair, head-to-head comparisons. All curves are shown with Loess-smoothing.

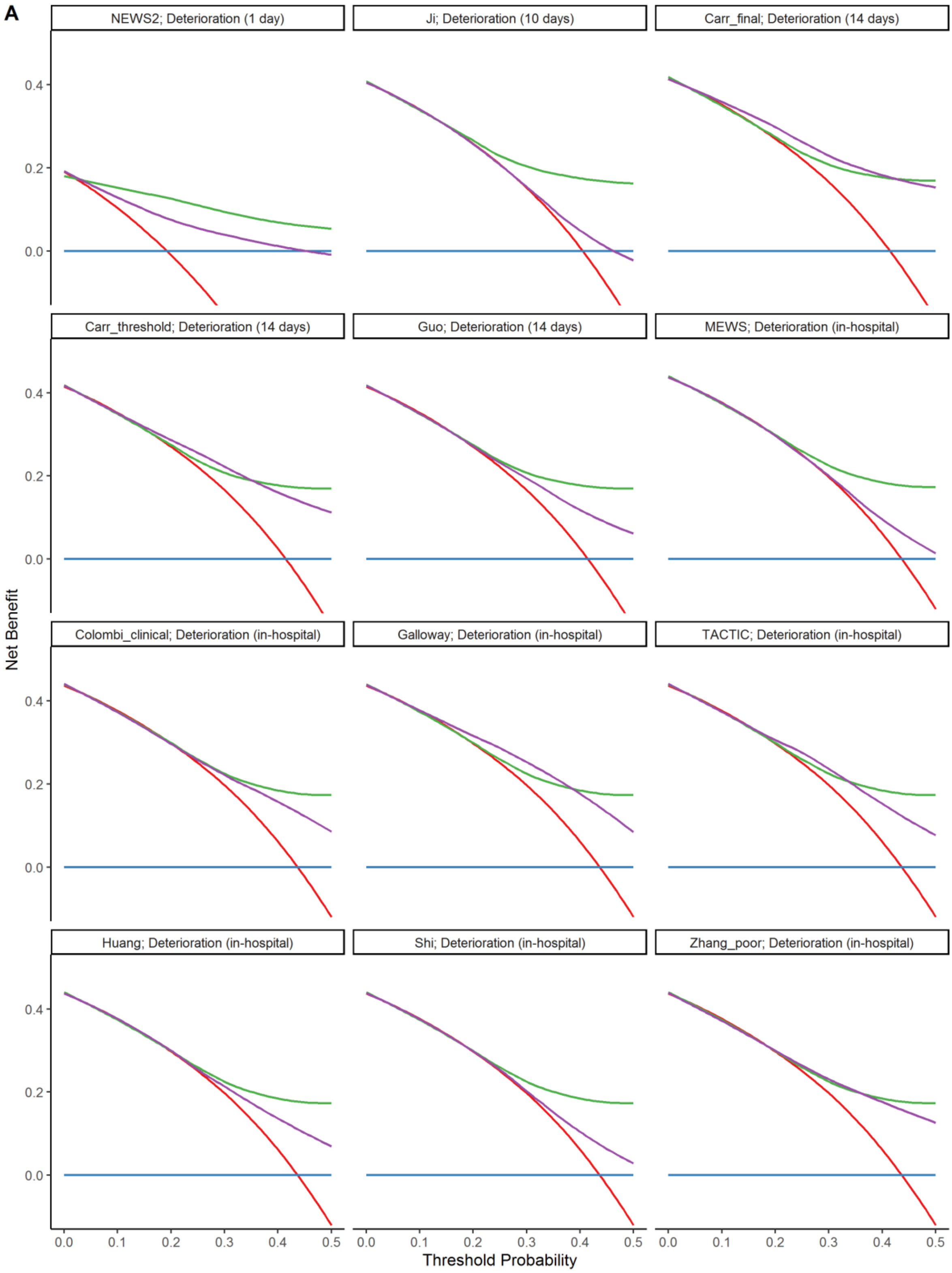
A
NEWS2; Deterioration (1 day)
Ji; Deterioration (10 days)
Carr_final; Deterioration (14 days)
Carr_threshold; Deterioration (14 days)
Guo; Deterioration (14 days)
MEWS; Deterioration (in-hospital)
Colombi_clinical; Deterioration (in-hospital)
Galloway; Deterioration (in-hospital)
TACTIC; Deterioration (in-hospital)
Huang; Deterioration (in-hospital)
Shi; Deterioration (in-hospital)
Zhang_poor; Deterioration (in-hospital)
Net Benefit
0.4
0.2
0.0
0.0
0.1
0.2
0.3
0.4
0.5
Threshold Probability

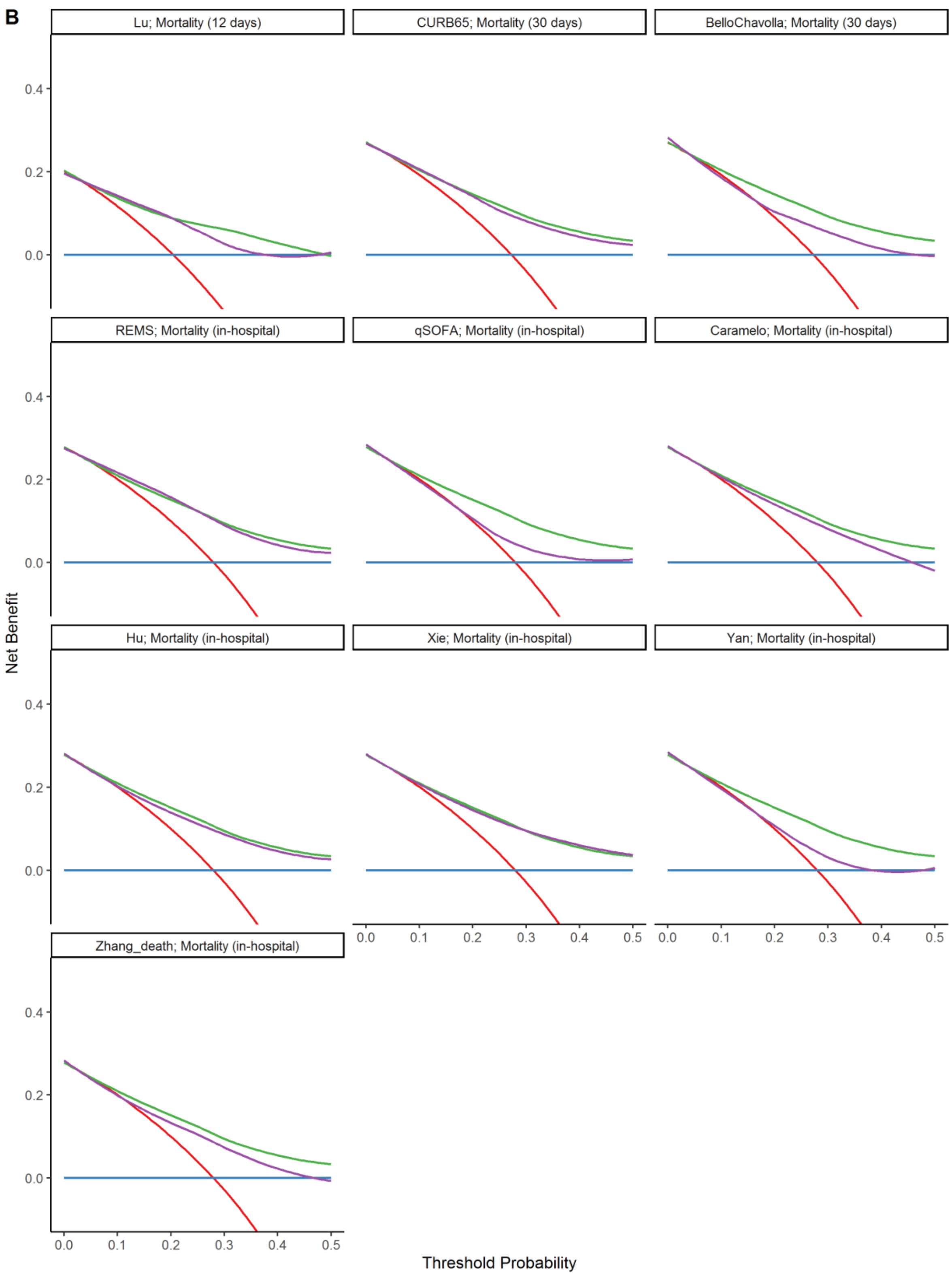
B
Lu; Mortality (12 days)
CURB65; Mortality (30 days)
BelloChavolla; Mortality (30 days)
REMS; Mortality (in-hospital)
qSOFA; Mortality (in-hospital)
Caramelo; Mortality (in-hospital)
Hu; Mortality (in-hospital)
Xie; Mortality (in-hospital)
Yan; Mortality (in-hospital)
Zhang_death; Mortality (in-hospital)
Net Benefit
Threshold Probability
0.4
0.2
0.0
0.0 0.1 0.2 0.3 0.4 0.5

Supplementary Figure 9: Restricted cubic splines plots showing associations between most discriminating univariable predictors of clinical deterioration and mortality and log odds of outcome, respectively.

Age was the most discriminating univariable predictor of in-hospital mortality, while oxygen saturation (SpO2) on air was the most discriminating predictor of in-hospital clinical deterioration. For both univariable predictors, associations appear approximately linear on the log odds scale.

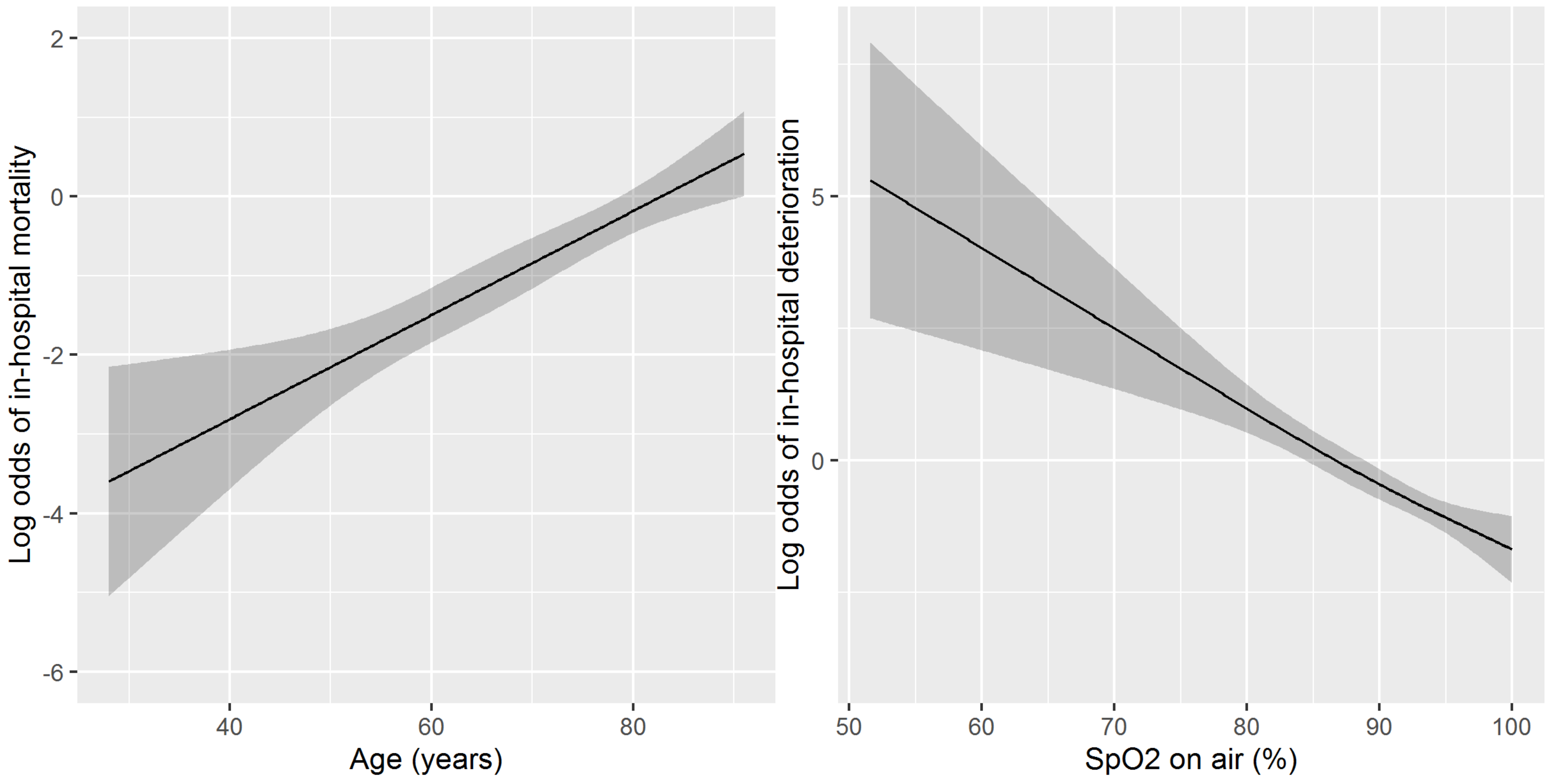

References

1. Wynants L, Calster B Van, Collins GS, Riley RD, Heinze G, Schuit E, Bonten MMJ, Damen JAA, Debray TPA, Vos M De, Dhiman P, Haller MC, Harhay MO, Henckaerts L, Kreuzberger N, Lohmann A, Luijken K, Ma J, Navarro CLA, Reitsma JB, Sergeant JC, Shi C, Skoetz N, Smits LJM, Snell KIE, Sperrin M, Spijker R, Steyerberg EW, Takada T, Kuijk SMJ van, et al. Prediction models for diagnosis and prognosis of covid-19: systematic review and critical appraisal. *BMJ* British Medical Journal Publishing Group; 2020; 369.
2. Bai X, Fang C, Zhou Y, Bai S, Liu Z, Chen Q, Xu Y, Xia T, Gong S, Xie X, Song D, Du R, Zhou C, Chen C, Nie D, Tu D, Zhang C, Liu X, Qin L, Chen W. Predicting COVID-19 malignant progression with AI techniques. *medRxiv* Cold Spring Harbor Laboratory Press; 2020; : 2020.03.20.20037325.
3. Barda N, Riesel D, Akriv A, Levi J, Finkel U, Yona G, Greenfeld D, Sheiba S, Somer J, Bachmat E, Rothblum GN, Shalit U, Netzer D, Balicer R, Dagan N. Performing risk stratification for COVID-19 when individual level data is not available, the experience of a large healthcare organization. *medRxiv* Cold Spring Harbor Laboratory Press; 2020; : 2020.04.23.20076976.
4. Chassagnon G, Vakalopoulou M, Battistella E, Christodoulidis S, Hoang-Thi T-N, Dangeard S, Deutsch E, Andre F, Guillo E, Halm N, Hajj S El, Bompard F, Neveu S, Hani C, Saab I, Campredon A, Koulakian H, Bennani S, Freche G, Barat M, Lombard A, Fournier L, Monnier H, Grand T, Gregory J, Nguyen Y, Khalil A, Mahdjoub E, Brillet P-Y, Ba ST, et al. Holistic AI-Driven Quantification, Staging and Prognosis of COVID-19 Pneumonia. *medRxiv* Cold Spring Harbor Laboratory Press; 2020; : 2020.04.17.20069187.
5. DAS A, Mishra S, Gopalan SS. Predicting community mortality risk due to CoVID-19 using machine learning and development of a prediction tool. *medRxiv* Cold Spring Harbor Laboratory Press; 2020; : 2020.04.27.20081794.
6. Gong J, Ou J, Qiu X, Jie Y, Chen Y, Yuan L, Cao J, Tan M, Xu W, Zheng F, Shi Y, Hu B. A Tool for Early Prediction of Severe Coronavirus Disease 2019 (COVID-19): A Multicenter Study Using the Risk Nomogram in Wuhan and Guangdong, China. *Clin. Infect. Dis.* Oxford University Press; 2020; 71: 833–840.
7. Jiang X, Coffee M, Bari A, Wang J, Jiang X, Huang J, Shi J, Dai J, Cai J, Zhang T, Wu Z, He G, Huang Y. Towards an Artificial Intelligence Framework for Data-Driven Prediction of Coronavirus Clinical Severity. *Comput. Mater. Contin.* 2020; 62: 537–551.
8. Levy TJ, Richardson S, Coppa K, Barnaby DP, McGinn T, Becker LB, Davidson KW, Cohen SL, Hirsch JS, Zanos T, Consortium N & MC-19 R. Development and Validation of a Survival Calculator for Hospitalized Patients with COVID-19. *medRxiv* Cold Spring Harbor Laboratory Press; 2020; : 2020.04.22.20075416.
9. Liang W, Liang H, Ou L, Chen B, Chen A, Li C, Li Y, Guan W, Sang L, Lu J, Xu Y, Chen G, Guo H, Guo J, Chen Z, Zhao Y, Li S, Zhang N, Zhong N, He J, COVID-19 for the CMTEG for. Development and Validation of a Clinical Risk Score to Predict the Occurrence of Critical Illness in Hospitalized Patients With COVID-19. *JAMA Intern. Med.* American Medical Association; 2020; 180: 1081.
10. Liu Q, Fang X, Tokuno S, Chung U, Chen X, Dai X, Liu X, Xu F, Wang B, Peng P. Prediction of the clinical outcome of COVID-19 patients using T lymphocyte subsets with 340 cases from Wuhan, China: a retrospective cohort study and a web visualization tool. *medRxiv* Cold Spring Harbor Laboratory Press; 2020; : 2020.04.06.20056127.
11. McRae MP, Simmons GW, Christodoulides NJ, Lu Z, Kang SK, Fenyo D, Alcorn T, Dapkins IP, Sharif I, Vurmaz D, Modak SS, Srinivasan K, Warhadpande S, Shrivastav R, McDevitt JT. Clinical Decision Support Tool and Rapid Point-of-Care Platform for Determining Disease Severity in Patients with COVID-19. *medRxiv* Cold Spring Harbor Laboratory Press; 2020; : 2020.04.16.20068411.
12. Pourhomayoun M, Shakibi M. Predicting Mortality Risk in Patients with COVID-19 Using Artificial Intelligence to Help Medical Decision-Making. *medRxiv* Cold Spring Harbor Laboratory Press; 2020; : 2020.03.30.20047308.
13. Qi X, Jiang Z, YU Q, Shao C, Zhang H, Yue H, Ma B, Wang Y, Liu C, Meng X, Huang S, Wang J, Xu D, Lei J, Xie G, Huang H, Yang J, Ji J, Pan H, Zou S, Ju S. Machine learning-based CT radiomics model for predicting hospital stay in patients with pneumonia associated with SARS-CoV-2 infection: A multicenter study. *medRxiv* Cold Spring Harbor Laboratory Press; 2020; : 2020.02.29.20029603.
14. Sarkar J, Chakrabarti P. A Machine Learning Model Reveals Older Age and Delayed Hospitalization as Predictors of Mortality in Patients with COVID-19. *medRxiv* Cold Spring Harbor Laboratory Press; 2020; : 2020.03.25.20043331.
15. Singh K, Valley TS, Tang S, Li BY, Kamran F, Sjoding MW, Wiens J, Otles E, Donnelly JP, Wei MY, McBride JP, Cao J, Penoza C, Ayanian JZ, Nallamothu BK. Evaluating a Widely Implemented Proprietary

Deterioration Index Model Among Hospitalized COVID-19 Patients. *medRxiv* Cold Spring Harbor Laboratory Press; 2020; : 2020.04.24.20079012.

16. Vaid A, Somani S, Russak AJ, Freitas JK De, Chaudhry FF, Paranjpe I, Johnson KW, Lee SJ, Miotto R, Zhao S, Beckmann N, Naik N, Arfer K, Kia A, Timsina P, Lala A, Paranjpe M, Glowe P, Golden E, Danieletto M, Singh M, Meyer D, O'Reilly PF, Huckins LH, Kovatch P, Finkelstein J, Freeman RM, Argulian E, Kasarskis A, Percha B, et al. Machine Learning to Predict Mortality and Critical Events in COVID-19 Positive New York City Patients. *medRxiv* Cold Spring Harbor Laboratory Press; 2020; : 2020.04.26.20073411.
17. Guillamet CV, Guillamet RV, Kramer AA, Maurer PM, Menke GA, Hill CL, Knaus WA. TOWARD A COVID-19 SCORE-RISK ASSESSMENTS AND REGISTRY. *medRxiv* Cold Spring Harbor Laboratory Press; 2020; : 2020.04.15.20066860.
18. Yuan M, Yin W, Tao Z, Tan W, Hu Y. Association of radiologic findings with mortality of patients infected with 2019 novel coronavirus in Wuhan, China. Schildgen O, editor. *PLoS One* Public Library of Science; 2020; 15: e0230548.
19. Zeng L, Li J, Liao M, Hua R, Huang P, Zhang M, Zhang Y, Shi Q, Xia Z, Ning X, Liu D, Mo J, Zhou Z, Li Z, Fu Y, Liao Y, Yuan J, Wang L, He Q, Liu L, Qiao K. Risk assessment of progression to severe conditions for patients with COVID-19 pneumonia: a single-center retrospective study. *medRxiv* Cold Spring Harbor Laboratory Press; 2020; : 2020.03.25.20043166.
