# Supplementary material for: Systematic evaluation and external validation of 22 prognostic models among hospitalised adults with COVID-19: an observational cohort study
Source: Eur Respir J. 2020 Dec 24;56(6):2003498. doi: 10.1183/13993003.03498-2020 (PMC7518075; doi:10.1183/13993003.03498-2020)
Supplement: Supplementary file 2 [file ERJ-03498-2020.Shareable.pdf]

SHAREABLE PDF

Systematic evaluation and external validation of 22 prognostic models among hospitalised adults with COVID-19: an observational cohort study

Rishi K. Gupta [1,2], Michael Marks [2,3], Thomas H.A. Samuels[2], Akish Luintel[2], Tommy Rampling[2], Humayra Chowdhury[2], Matteo Quartagno[4], Arjun Nair[2], Marc Lipman [5], Ibrahim Abubakar [1], Maarten van Smeden [6], Wai Keong Wong[2], Bryan Williams[7,8] and Mahdad Noursadeghi [2,9], on behalf of The UCLH COVID-19 Reporting Group[10]

Affiliations: [1]Institute for Global Health, University College London, London, UK. [2]University College London Hospitals NHS Trust, London, UK. [3]Clinical Research Dept, Faculty of Infectious and Tropical Diseases, London School of Hygiene and Tropical Medicine, London, UK. [4]MRC Clinical Trials Unit, Institute of Clinical Trials and Methodology, University College London, London, UK. [5]UCL Respiratory, Division of Medicine, University College London, London, UK. [6]Julius Center for Health Sciences and Primary Care, University Medical Center Utrecht, Utrecht University, Utrecht, The Netherlands. [7]NIHR University College London Hospitals Biomedical Research Centre, London, UK. [8]University College London, London, UK. [9]Division of Infection and Immunity, University College London, London, UK. [10]Members of the UCLH COVID-19 Reporting Group are listed in the acknowledgements section.

Correspondence: Mahdad Noursadeghi, Division of Infection and Immunity, Cruciform Building, University College London, London WC1E 6BT, UK. E-mail: m.noursadeghi@ucl.ac.uk

@ERSpublications
Oxygen saturation on room air and patient age are strong predictors of deterioration and mortality, respectively, among hospitalised adults with COVID-19. None of the 22 prognostic models evaluated in this study adds incremental value to these univariable predictors. https://bit.ly/2Hg24TO

Cite this article as: Gupta RK, Marks M, Samuels THA, *et al*. Systematic evaluation and external validation of 22 prognostic models among hospitalised adults with COVID-19: an observational cohort study. *Eur Respir J* 2020; 56: 2003498 [https://doi.org/10.1183/13993003.03498-2020].

This single-page version can be shared freely online.

ABSTRACT The number of proposed prognostic models for coronavirus disease 2019 (COVID-19) is growing rapidly, but it is unknown whether any are suitable for widespread clinical implementation.

We independently externally validated the performance of candidate prognostic models, identified through a living systematic review, among consecutive adults admitted to hospital with a final diagnosis of COVID-19. We reconstructed candidate models as per original descriptions and evaluated performance for their original intended outcomes using predictors measured at the time of admission. We assessed discrimination, calibration and net benefit, compared to the default strategies of treating all and no patients, and against the most discriminating predictors in univariable analyses.

We tested 22 candidate prognostic models among 411 participants with COVID-19, of whom 180 (43.8%) and 115 (28.0%) met the endpoints of clinical deterioration and mortality, respectively. Highest areas under receiver operating characteristic (AUROC) curves were achieved by the NEWS2 score for prediction of deterioration over 24 h (0.78, 95% CI 0.73–0.83), and a novel model for prediction of deterioration <14 days from admission (0.78, 95% CI 0.74–0.82). The most discriminating univariable predictors were admission oxygen saturation on room air for in-hospital deterioration (AUROC 0.76, 95%

Copyright ©ERS 2020. This version is distributed under the terms of the Creative Commons Attribution Licence 4.0.

CI 0.71–0.81), and age for in-hospital mortality (AUROC 0.76, 95% CI 0.71–0.81). No prognostic model demonstrated consistently higher net benefit than these univariable predictors, across a range of threshold probabilities.

Admission oxygen saturation on room air and patient age are strong predictors of deterioration and mortality among hospitalised adults with COVID-19, respectively. None of the prognostic models evaluated here offered incremental value for patient stratification to these univariable predictors.
